# Biogenic Selenium Nanoparticles: A Fine Characterization to Unveil Their Thermodynamic Stability

**DOI:** 10.3390/nano11051195

**Published:** 2021-05-01

**Authors:** Elena Piacenza, Alessandro Presentato, Francesco Ferrante, Giuseppe Cavallaro, Rosa Alduina, Delia F. Chillura Martino

**Affiliations:** 1 Department of Biological, Chemical, and Pharmaceutical Sciences and Technologies (STEBICEF), University of Palermo, Viale delle Scienze Ed. 16, 90128 Palermo, Italy; valeria.alduina@unipa.it (R.A.); delia.chilluramartino@unipa.it (D.F.C.M.); 2 Department of Physics and Chemistry “Emilio Segrè” (DIFC), University of Palermo, Viale delle Scienze Ed. 17, 90128 Palermo, Italy; francesco.ferrante@unipa.it (F.F.); giuseppe.cavallaro@unipa.it (G.C.)

**Keywords:** biogenic selenium nanoparticles, thermodynamic stability, selenium nanorods, *Micrococcus*, FTIR spectroscopy, DFT calculations, multivariate statistical analysis

## Abstract

Among the plethora of available metal(loid) nanomaterials (NMs), those containing selenium are interesting from an applicative perspective, due to their high biocompatibility. Microorganisms capable of coping with toxic Se-oxyanions generate mostly Se nanoparticles (SeNPs), representing an ideal and green alternative over the chemogenic synthesis to obtain thermodynamically stable NMs. However, their structural characterization, in terms of biomolecules and interactions stabilizing the biogenic colloidal solution, is still a black hole that impairs the exploitation of biogenic SeNP full potential. Here, spherical and thermodynamically stable SeNPs were produced by a metal(loid) tolerant *Micrococcus* sp. Structural characterization obtained by Scanning Electron Microscopy (SEM) revealed that these SeNPs were surrounded by an organic material that contributed the most to their electrosteric stabilization, as indicated by Zeta (ζ) potential measurements. Proteins were strongly adsorbed on the SeNP surface, while lipids, polysaccharides, and nucleic acids more loosely interacted with SeNMs as highlighted by Fourier Transform Infrared Spectroscopy (FTIR) and overall supported by multivariate statistical analysis. Nevertheless, all these contributors were fundamental to maintain SeNPs stable, as, upon washing, the NM-containing extract showed the arising of aggregated SeNPs alongside Se nanorods (SeNRs). Besides, Density Functional Theory (DFT) calculation unveiled how thiol-containing molecules appeared to play a role in SeO_3_^2−^ bioreduction, stress oxidative response, and SeNP stabilization.

## 1. Introduction

The rapid and exponential growth of nanotechnology during the last 40 years has led to the development of many synthetic procedures to generate nanomaterials (NMs) featuring different sizes, shapes, and compositions for various (bio)technological purposes [1]. Selenium nanostructures (SeNSs) have gained technological interest, due to their physical-chemical versatility [2] and their efficiency as components of renewable energy production devices, constituting an important alternative over fossil-fuel technologies [3]. Besides, SeNMs feature high biocompatibility, being Se an essential micronutrient for living organisms, favoring SeNS (bio)technological and biomedical applications compared to other metal-based NMs [4]. Since the early 2000s, we witnessed a constant expansion of physical and chemogenic procedures devoted to the mass-production of high-quality SeNSs [2]. However, most of these approaches rely on dangerous operational conditions and the use of toxic substances [2] that endanger both human health and the environment. Thus, alternative synthetic methodologies are needed to produce green SeNMs, among which, those based on environmental-friendly bacteria and biocompatible chemical reagents are the most appealing [5]. In this regard, a variety of bacteria showed the ability to transform micro-essential yet toxic Se-containing oxyanions (i.e., selenate (SeO_4_^2−^) and selenite (SeO_3_^2−^)) into their less bioavailable elemental forms (Se^0^) generating either intra- or extra-cellular NMs of great technological and economical values [5,6]. To date, the most common process elicited by microorganisms to cope with SeO_3_^2−^ relies on Painter-type reactions. Following this mechanism, thiol (RSH)-containing molecules (glutathione (GSH), mycothiol (MSH), and bacillithiol (BSH)) partially reduce SeO_3_^2−^ through their cysteine moieties as a first stress-response mechanism [7,8,9]:

3RSH + 6SeO_3_^2−^ + 4H^+^ → 3RSSeSR + 2O_2_^−^ + 5H_2_O (1)

The as-produced RSSeSR intermediate then undergoes a series of transformations mediated by a NAD(P)H-dependent enzyme (i.e., glutathione reductase) that allows the complete reduction of Se oxyanion to Se^0^ [9]:

3RSSeRS + 3NADP(H) → 3RSSe^−^ + 3RSH + 3NAD(P)^+^ (2)

3RSSe^−^ + H^+^ → 3RSH^−^ + 3Se^0^ (3)

A major applicative advantage of biogenic SeNMs over those of chemogenic synthesis implies the spontaneous generation of thermodynamically stable yet structurally diverse SeNSs, hence ruling out the need for post-production treatments before their use [10]. However, the practical application of biogenic SeNMs is prevented by a lack of understanding of their structure-to-property relationships, compared to other more studied nanotechnological products (e.g., silver or gold NMs).

In the present study, new insights on the structure of SeNSs, and their behavior with respect to the physical and chemical proximities, were put forward, focusing on SeNPs produced by the environmental isolate *Micrococcus* sp., incubated with sodium selenite (Na_2_SeO_3_). The as-obtained SeNPs were characterized in terms of their size, morphology, and surrounding environment, through Scanning Electron Microscopy (SEM), Zeta (ζ) potential measurements, and Fourier Transform Infrared spectroscopy in Attenuated Total Reflectance (ATR-FTIR) mode, to shed light on the reason behind the enhanced thermodynamic stability of biogenic SeNMs. Moreover, a multivariate statistical analysis (i.e., Principal Component Analysis, PCA) was used to support the results obtained through ATR-FTIR spectroscopy, focusing on biomolecules interacting the most with the biogenic SeNSs. Finally, given the complexity of the systems analyzed, Density Functional Theory (DFT) calculations were carried out on SeNP models and RSH-containing or deriving molecules to confirm the results obtained from the experimental measurements, gaining new information on the NP structure.

## 2. Materials and Methods

### 2.1. Materials

All the reagents were purchased from Sigma-Aldrich^®^ (Milan, Italy), except for deuterium oxide (D_2_O), which was obtained from Cambridge Isotope Laboratories, Inc. (Tewksbury, MA, USA). Crystalline Silicon wafers (type N/Phos, size 100 mm), Specimen Aluminum stubs, and folded capillary Zeta cell were purchased from University WAFER (Milan, Italy), TED PELLA, INC. (Milan, Italy) and Malvern Instruments (Malvern, UK), respectively.

### 2.2. Bacterial Growth and Loss of Thiol Pool as a Consequence of SeO_3_^2−^ Bioprocessing

The bacterial strain *Micrococcus* sp., previously isolated from metal(loid)-rich Japanese wallpapers [11], was chosen for SeNP synthesis, due to its tolerance towards metal(loid) compounds. The bacterial strain was pre-cultured in Tryptic Soy Broth (TSB)-rich medium for 72 h at 30 °C with shaking (180 rpm), being afterwards inoculated (1% volume/volume (*v*/*v*)) in M9 minimal salts medium amended with 0.5% (*w*/*v*) glucose as a carbon and energy source, as well as in the presence/absence of 0.5 mM Na_2_SeO_3_ for 120 h. The capability of *Micrococcus* sp. to thrive under selenite (SeO_3_^2−^) stress was evaluated monitoring its growth and oxyanion removal over the time. The bacterial growth was assessed every 24 h through spot plate count method [12]; the data are reported as the average (*n* = 3) logarithm of colony forming unit (CFU) mL^−1^ (log_10_ (CFUmL^−1^)) with standard deviation (SD).

Aliquots (1 mL) of bacterial cultures were recovered every 24 h of growth to monitor RSH oxidation, as described by Turner et al. [13]. The absorbance of the suspensions containing the RSH- 5,5-dithio-bis-2-nitrobenzoic acid (DTNB) complex was read at 412 nm using a Beckman Coulter DU 800 (Beckman Coulter Life Sciences, Milan, Italy). RSH concentration was determined and normalized for the total amount of cell proteins following the procedure reported by Piacenza and colleagues [14]; data are reported as average values (*n* = 3) of loss of RSH from the original pool with SD.

### 2.3. Biogenic Selenium Nanoparticles Preparation

The biogenic SeNP extracts (herein indicated as Bio SeNP extract) were recovered from *Micrococcus* sp. cells incubated for 120 h with SeO_3_^2−^ following the procedure described by Piacenza et al. [14].

To study the role of biomolecules within biogenic extracts in the thermodynamic stabilization of SeNPs, the obtained NP suspension was centrifuged (14000× *g* for 10 min) and resuspended in ddH_2_O (henceforth referred to as Bio SeNP extract_w). The collected supernatants containing the Organic Material (OM) surrounding SeNPs was recovered for further analysis.

For clarity, the nomenclature and description of the analyzed samples are reported in Table 1.

### 2.4. SeNP Characterization

The morphology and size of biogenic SeNPs were evaluated through Scanning Electron Microscopy (SEM), using a FEG-SEM FEI versa 3D^TM^ microscope (Thermo Fischer Scientific Electron Microscopy Solutions, Hillsboro, OR, USA) at an accelerating voltage of 10 kV. Briefly, 5 µl aliquots of Bio SeNP extract and Bio SeNP extract_w was deposited onto 1 cm × 1 cm Crystalline Silicon wafers, mounted on specimen aluminum stubs, and air-dried prior their visualization [15]. The actual size of SeNMs was determined using ImageJ software (1.50i, National Institute of Health, Rockville Pike Bethesda, MD, USA) by measuring 100 randomly chosen NPs and NRs for each analyzed sample.

The surface charge was monitored to obtain indications regarding electrostatic interactions potentially occurring between the components of Bio SeNP extract, OM, and Bio SeNP extract_w. Zeta (ζ) potential measurements were performed, under isothermal conditions (T = 25 °C), three times (100 scans each, acquisition time 30 s) on 1 mL aliquots using a Zen 3600 Zetasizer Nano ZS ^TM^ (Malvern Instruments, Malvern, UK). The experimental data are reported as average value (*n* = 3) with standard deviation.

### 2.5. Fourier Transform Infrared Spectroscopy in Attenuated Totale Reflectance (ATR-FITR) Mode

ATR-FTIR spectra were collected for all suspensions of Table 1 by using an FTIR Bruker Vertex70 Advanced Research FTIR Spectrometer (Billerica, MA, USA) equipped with a Platinum ATR and a diamond crystal. The spectra were recorded in the 70–4000 cm^−1^ range with a lateral resolution of 2 cm^−1^ and 200 scans and were analyzed through OPUS7.5 (Bruker Instruments) and OriginPro 2016 software. Spectral deconvolution, when applied, alongside peak area determination, were performed by using the curve fitting method package of OriginPro 2016 software.

The percentage area of diverse amide I components related to protein secondary structures (i.e., β-antiparallel, β-turn, α-helix, and β-sheet) was estimated as reported by Byler and colleagues [16].

### 2.6. Density Functional Theory (DFT) Calculations

Since one of the most accredited mechanism of SeO_3_^2−^ reduction elicited by microorganisms involves RSH chemistry [17], the geometries and harmonic IR spectra of systems where L-cysteine and its derivatives interact with one Se_8_ unit (Figure S1), as well as those of the isolated molecules, were calculated within the Density Functional Theory framework. All calculations were performed by means of the Gaussian 16 program [18], using the hybrid Becke 3-parameter Lee–Yang–Parr (B3LYP) exchange-correlation functional [19,20] joined with the correlation-consistent polarized valence double zeta (cc-pvDZ) basis set [21]. In order to achieve a reliable description of the interaction between L-cysteine (and derivatives) and the Se_8_ unit, it was deemed necessary to modify the exchange-correlation functional by including the treatment of dispersion interactions. This was afforded in the present investigation by employing the third version of the empirical correction proposed by Grimme [22].

### 2.7. Multivariate Statistical Analysis of ATR-FTIR Spectra

Principal Component Analysis (PCA) was chosen as multivariate statistical approach to shed some light regarding the contribution of diverse biomolecules in the thermodynamic stabilization of biogenic SeNPs. Thus, based on the assignment of the detected IR absorption bands (Tables S3 and S4), contributions typical of lipids, proteins, or polysaccharides were selected to perform PCA. Specifically, PCA was independently carried out for lipids, proteins, and polysaccharides by constructing a data matrix considering the IR contributions as variables and the corresponding areas for each sample listed in Table 1 as observations. After PCs were identified, they were derived inside the final clusters to find an optimal orthogonal linear projection on a *d*-dimensional subspace retaining the maximum of the original information [11]. Once the most important variables for each biomolecular class were determined by analyzing both the resulting clustering of observations (PCA score plot) and variable vectors (PCA loading plot), a new PCA was carried out by combining these contributions together to obtain an overview of the biomolecules’ involvement in the samples under study. All PCA were performed by using the multivariate statistical analysis package of OriginPro 2016 software.

## 3. Results and Discussion

### 3.1. Bacterial Tolerance towards SeO_3_^2−^ Oxyanion

*Micrococcus* sp. cells were negatively affected by the metabolic controlled growth conditions (i.e., exploitation of glucose as carbon and energy sources), as highlighted by cell death events occurring after 48 h of bacterial incubation (Figure S2a), which is in line with previous observations [12]. This aspect may be ascribed to an excess (0.5% *w*/*v*) of glucose that can determine the arrest of the tricarboxylic acid cycle, resulting in the accumulation of pyruvate in the cytoplasm [23]. The latter can be oxidized into acetic acid, whose catabolism can stimulate the murein hydrolase activity, leading to cell lysis and death [24]. An even more drastic effect on the growth extent of *Micrococcus* sp. was observed when SeO_3_^2−^ was added, as no active growth of bacterial cells was detected (Figure S2a), likely due to the toxicity exerted by this oxyanion. To date, the most accredited biochemical mechanism of SeO_3_^2−^ toxicity is the generation, during the oxyanion biotransformation, of reactive oxygen species (ROS; e.g., superoxide anion (O_2_^−^), hydrogen peroxide (H_2_O_2_), and hydroxyl radical (OH^−^)) causing oxidative stress, such as irreversible damage to cell proteins, lipids, and DNA [9,17,25], which will be further discussed.

### 3.2. Characterization of Biogenic SeNPs

**Figure 1:**
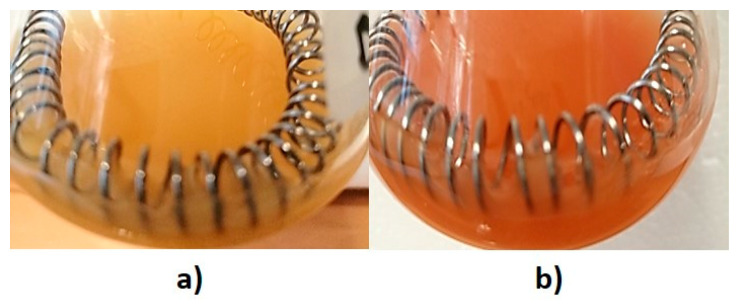
*Micrococcus* sp. culture incubated for 120-h in M9 medium supplied with (**a**) glucose or (**b**) glucose and Na_2_SeO_3_. The color variation indicates the formation of elemental Se (Se^0^).

The reduction of SeO_3_^2−^ into Se^0^ was macroscopically visible through a color change of *Micrococcus* sp. cultures from dark yellow to orange-red (Figure 1). This phenomenon is due to the development of exciton resonance within Se atoms, which determines the rising of unique optical properties as a function of the size and shape of SeNMs, as well as their surrounding environment [26].

**Figure 2:**
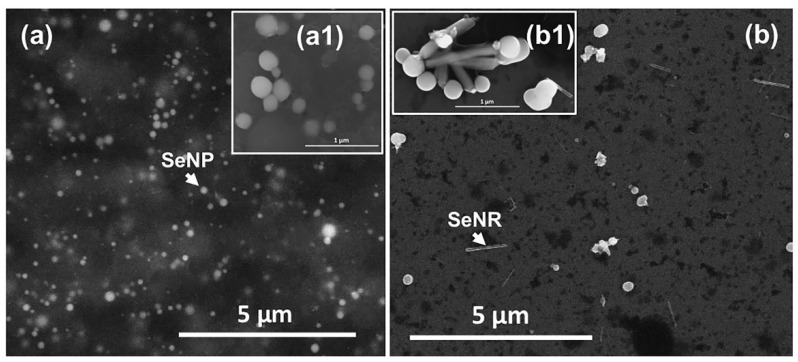
: Scanning electron micrographs of (**a**,**a1**) Bio SeNP extract and (**b**,**b1**) Bio SeNP extract_w. Arrow heads indicate the occurrence of SeNPs and SeNRs, while the inlets (**a1**) and (**b1**) displayed the observed SeNMs at high magnification.

*Micrococcus* sp. produced spherical and highly regular SeNPs, which were surrounded or embedded by a material with low electron emission yield deriving from bacterial cells (Figure 2a,a1), being in line with most of the previous reports on biogenic NMs [10,27].

Although biogenic SeNPs featured an average diameter of 149 ± 34 nm, which is beyond the classical nanorange (1–100 nm), the arising of unique physical-chemical (i.e., exciton resonance) properties of these NMs allow their consideration as NPs *per sè* [28]. A fairly high polydispersity in size was observed within Bio SeNP extract (Figure 2a), reasonably attributable to the uneven distribution of SeO_3_^2−^ within *Micrococcus* sp. cells, which may determine diverse oxyanion reduction and NP production rates [29]. Nevertheless, the average diameter measured for biogenic SeNPs under study agreed with most reports available to date [5,6]. The bacterial derived material, or organic material (OM), surrounding SeNPs (Figure 2a,a1) appeared to be responsible for the thermodynamic stabilization of these NMs [10,30], as any aggregation phenomena were not observed by SEM imaging upon air-drying of the aqueous Bio SeNP extract on the Si-slide (Figure 2a,a1). This hypothesis was firstly confirmed by the identification of big SeNPs (293 ± 89 nm), SeNP aggregates, and even the formation of Se nanorods (SeNRs; 755 ± 150 nm) upon a single washing step of the original biogenic extract (Figure 2b,b1). This procedure may have altered the electrosteric barrier represented by the OM adsorbed onto SeNP surfaces [30,31], increasing their energy and reactivity, causing, in turn, their aggregation in larger NMs to overcome their thermodynamic instability. Additionally, the loss of OM might have caused SeNPs to both partially dissolve and release Se atoms, due to their Rayleigh instability, and aggregate forming linear NP chains (i.e., oriented attachment phenomenon) [32]. Consequently, to these processes, Se free atoms and those within the NP chains likely underwent a phase transformation from amorphous (red) Se (*a*-Se) to the more stable trigonal (grey) Se (*t*-Se). The latter consists of one dimensional (1D) infinite helical chains, featuring high anisotropic crystal structure, which determines *t*-Se preference to grow along one axis, forming SeNRs, to lower their Gibbs free energy [2,31,32]. Hence, SeNRs detected within Bio SeNP extract_w (Figure 2b,b1) were likely obtained through both (i) the crystallization of pre-existing and preferentially oriented amorphous SeNPs and (ii) the formation of *t*-Se nucleation seeds from Se atoms released by dissolved *a*-SeNPs [32].

The nature of SeNP stabilization was first evaluated by monitoring the surface charge of the samples under investigation, whose results are displayed in Table 2.

The detection of negative ζ potential values for all the suspensions (Table 2) indicated the presence of electrostatic repulsive forces (hence at least an electrostatic stabilization) between their components. Since Se does not have a net charge in its elemental valence state, the measured surface charges can be attributable to the adsorption of ions/charged functional groups present in solution onto the surface of SeNPs, either during or after the NP synthesis and assembly [33,34]. In this regard, the obtained negative surface charges may derive from diverse biomolecules present in the OM surrounding SeNPs, such as proteins, (phospho)lipids, nucleic acids, and polysaccharides [10], being in line with the results reported for other biogenic SeNMs [30,31,35,36,37]. This hypothesis was supported by the less negative ζ potential value observed for Bio SeNP extract_w than the original extract, whose surface charge was instead comparable to that of the recovered supernatant (i.e., OM) (Table 2). Indeed, in colloidal suspensions, such as those investigated here, ions/functional groups form the so-called Electrical Double Layer (EDL), which comprises two layers of ions/charged functional groups more tightly (Stern) or less firmly (Gouy layer) associated with the NPs [38]. Thus, the washing treatment could have partially removed the excess of functional groups less tightly adsorbed onto SeNP surfaces, decreasing the repulsion forces within these suspensions, as also suggested by the detection large NPs, aggregates, and SeNRs (Figure 2b,b1). This observation also suggested the existence of a multi-layer structure surrounding biogenic SeNPs, whose nature depends on the functional groups, hence biomolecules, deriving from bacterial cells. Furthermore, the small difference in ζ potential between the unwashed and washed Bio SeNP extracts can indicate an equilibrium between the biomolecules within the OM interacting with NPs and those present in solution [14,31].

### 3.3. Biomolecules Involved in SeO_3_^2−^ Bioprocessing and SeNP Stabilization

**Figure 3:**
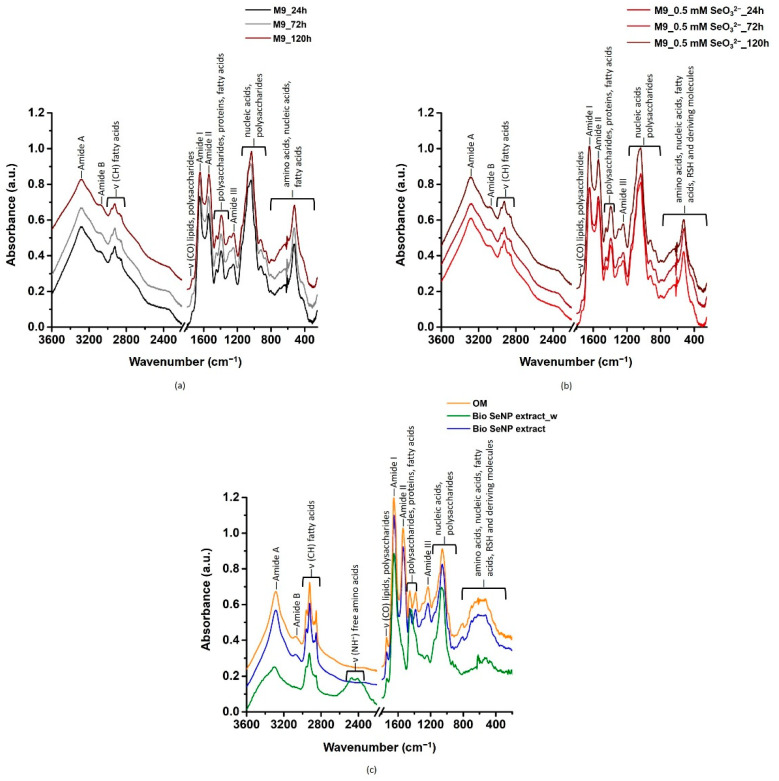
ATR-FTIR spectra of *Micrococcus* sp. cells exposed for 24, 72, and 120 h to (**a**) glucose or (**b**) glucose and SeO_3_^2−^, and (**c**) the recovered biogenic extracts and OM. For clarity, the spectra were offset of 0.1 a.u.

ATR-FTIR spectroscopy was carried out on both *Micrococcus* sp. unchallenged cells and those exposed to SeO_3_^2−^, as well as biogenic SeNP extracts, to unveil functional groups and, when possible, biomolecules likely involved in both the synthesis and stabilization of SeNPs (Figure 3). Full band assignments (as observed maxima) are reported in Tables S3 and S4.

Overall, ATR-FTIR spectra collected for *Micrococcus* sp. SeO_3_^2−^-free culture and those incubated with the oxyanion showed IR signals derived from lipids, proteins, polysaccharides, and nucleic acids (Figure 3a,b; Table S3). Biogenic SeNP extracts and the OM featured vibrational modes attributable to the same biomolecular classes (Figure 3c; Table S4), which acted as stabilizing agents for NPs, as also suggested by SEM imaging (Figure 2) and surface charge measurements (Table 2). In the next sections, IR absorption modes of each class of biomolecules will be disserted.

#### 3.3.1. Lipids

Lipid contributions were observed as high-intensity IR signals detected in the 2950–2850 cm^−1^ (Figure 3), which corresponded to asymmetric and symmetric -CH stretching vibrations mainly of methylene groups (-CH_2_) of fatty acid aliphatic chains (Tables S3 and S4) [30,39,40,41]. Moreover, weak IR absorption bands centered at ca. 1740 cm^−1^ and 720 cm^−1^ were identified as carbonyl (-C=O) stretching and -CH_2_ rocking vibrations of ester moieties and fatty acid chains respectively, (Figure 3; Tables S3 and S4) typically found in bacterial lipids and triglycerides [30,39,40,41]. The detection of lipids and derivatives within biogenic SeNP extracts and the OM indicates the involvement of these biomolecules in the NP stabilization, as they can be responsible for the arising of relevant steric and/or electrostatic (if a charge distribution is present) repulsive interactions within the extracts [10]. Additional IR signals deriving from -CH_2_ and methyl (-CH_3_) bending and deformation modes (shoulder at ca. 1468 cm^−1^) alongside the asymmetric carboxyl (-COO^−^) stretching vibration (ca. 1390 cm^−1^) of lipids were detected in ATR-FTIR spectra of *Micrococcus* sp. cells, being however absent in the biogenic extracts and the OM (Tables S3 and S4). This observation can be due to the partial loss of lipids during the extraction procedure of biogenic SeNPs.

PCA performed considering the relevant lipid IR contributions accounted for 93% of the original information and three main PCs were identified (PC1 = 56.3%, PC2 = 23.8%, and PC3 = 12.8%) (Figure S3a,b). PC1 retained most data and separated mainly for asymmetric and symmetric -CH stretching vibrations of fatty acids, which were strongly interdependent and comparable for all samples, except for Bio SeNP extract_w (Figure S3b and Table S4). IR vibrations of -C=O stretching, -CH_3_ deformation, -CO rocking, and symmetric -PO_2_^−^ influenced the most PC2 and PC3, causing a finer separation of the analyzed samples (Figure S3a,b). Thus, the PCA score plot showed the clustering of (i) *Micrococcus* sp. SeO_3_^2−^-free culture incubated for 24 and 72 h, and (ii) bacterial cells exposed for 72 and 120 h to the oxyanion alongside the Bio SeNP extract, while the other samples stood alone, being Bio SeNP extract_w the most distant one (Figure S3a). These results reflect the growth profiles of *Micrococcus* sp. cells, as an active growth was observed only for unchallenged culture up to 48 h of incubation (Figure S2a). The groups represented by bacterial cells incubated for either 24 h (SeO_3_^2−^) or 120 h (unchallenged) (Figure S3a) can depend on the initial oxyanion deriving stress and cell death events, respectively (Figure S2a). The intermediate localization, in terms of lipid vibrational modes, of the OM between the two observed clusters, the distance of Bio SeNP extract_w from the other samples (Figure S3a), together with both the lower relative areas and the disappearance of some lipid IR signals in the latter (Figure 3c and Table S4), indicated that these biomolecules may be loosely adsorbed onto the SeNP surface [30].

#### 3.3.2. Proteins

Strong IR absorption bands typical of amide A (ca. 3285 cm^−1^), amide B (ca. 3070 cm^−1^), amide I (ca. 1650 cm^−1^), and amide II (ca. 1540 cm^−1^), alongside low-intensity amide III (1260–1240 cm^−1^) (Figure 3, Tables S3 and S4), highlighted the crucial contribution of proteins in all the analyzed samples [30,35,36,39,40,41,42,43,44,45,46,47,48,49,50], although important differences were observed. Specifically, both amide I and amide II contributions underwent modifications when *Micrococcus* sp. cells were incubated with SeO_3_^2−^ (Table S3), indicating protein participation in the bioprocessing of this oxyanion. The amide I spectral region (ca. 1645 cm^−1^) was highly convoluted in the case of bacterial cells exposed to SeO_3_^2−^, while *Micrococcus* sp. SeO_3_^2−^-free culture displayed a prominent peak at ca. 1652 cm^−1^ and a shoulder at ca. 1640 cm^−1^ (Figure 3a and Table S3). These contributions were earlier reported for α-helix (ca. 1650 cm^−1^) and β-sheet (ca. 1640 cm^−1^) secondary structures [51]. An opposite behavior was noted for the amide II band (ca. 1540 cm^−1^), which showed, for bacterial cells exposed to the oxyanion, two shoulders centered at 1560 and 1515 cm^−1^ (Table S3). The detection of these IR signals in both biogenic SeNP extracts and OM (Figure 3c and Table S4) indicated the crucial role of *Micrococcus* sp. proteins not only in SeO_3_^2−^ reduction but also in SeNP assembly and stabilization [30,52]. In this regard, proteins adsorbed onto the SeNP surface can contribute as both electrostatic and steric stabilizers for biogenic NPs [10]. This hypothesis was supported by the disappearance (amide B) or the modification, in terms of intensity (amide III) and shape (amide II) of protein vibrational modes in the Bio SeNP extract_w and the arising of absorption bands in the 2475–2410 cm^−1^ spectral region, which are typical of -NH^+^ stretching vibration of free amino acids (Figure 3c and Table S4) [53]. Thus, upon washing of the Bio SeNP extract, part of the original proteins may be removed [40] and/or altered in their physical-chemical structure, modifying the chemical proximity of SeNPs. The less negative ζ potential value noted for Bio SeNP extract_w than the original extract (Table 2), which may be due to both (i) the partial removal of negatively charged biomolecules and (ii) the arising of -NH^+^ stretching vibration (Figure 3c), as well as the detection of NM aggregates morphology shift from NP to NR (Figure 2) further corroborated the role of proteins as electrosteric stabilizers. 

Spectral deconvolutions in the 1780–1480 cm^−1^ region (Figure S4) underlined variations in terms of peaks, width, and area between *Micrococcus* sp. SeO_3_^2−^-free culture and Se-containing samples (Tables S5–S7), likely due to both the presence of structurally diverse proteins and their different adsorption onto the SeNP surface. Particularly, vibrational modes typical of α-helix (1660–1650 cm^−1^) were identified for all samples, while β-antiparallel (1690–1680 cm^−1^), β-turn (ca. 1670 cm^−1^), and β-sheet (1640–1630 cm^−1^) motifs [51] were relevantly observed for bacterial cells incubated with the oxyanion, the biogenic extracts, and OM (Table 3 and Table S5–S7).

Unchallenged bacterial cells showed the lowest amide I calculated areas (Table 3), further confirming the importance of proteins for SeO_3_^2−^ bioprocessing, SeNP assembly, and stabilization [10,30,52]. Moreover, the higher percentages of β-strand structures than α-helix motifs in Se-containing samples (Table 3) is typical of several Se-interacting and reducing proteins or enzymes (e.g., glutaredoxins, mycoredoxins, and the selenium-binding protein SeBP), due to the propension of cysteine residues to form β-strand conformations [54,55,56,57,58]. In line with this, Geng and colleagues reported on the ability of SeO_3_^2−^ to cause conformation changes (increase in β-sheet motifs) in a recombinant human arsenic(III) methyltransferase upon interaction of Se^4+^ with sulfhydryl groups of cysteine residues [59]. Similarly, these motifs were highly represented within SeNP extracts recovered from several *Bacillus* strains [35,36,42,43,50], *Acinetobacter* sp. SW30 [60], and *Lactobacillus casei* 393 [61]. However, protein contributions exclusively attributable to α-helix motifs were detected when *Providencia rettgeri* HF16-A, *B. safensis* JT-B5T, *Bacillus* sp., and *Streptomyces minutiscleroticus* M10A62 were exploited as cell factories to produce SeNPs [40,41,44,48]. Thus, variations observed between SeNP extracts recovered from diverse microorganisms could rely on (i) the intrinsic differences between bacterial strains and strategies elicited for SeO_3_^2−^ bioconversion, (ii) SeNP localization that could lead to the presence of diverse proteins interacting with NPs, and (iii) the extraction procedure used, which can modify the physical-chemical structure of the OM surrounding SeNPs.

Bio SeNP extract_w showed the lowest protein secondary structure variability featuring only α-helix and β-sheet motifs (Table 3 and Table S7), being in line with previous reports on SeNPs produced by *Azospirillum thiophilum* VKM B-2513 and washed with water [30]. Particularly, the large content of α-helix motifs observed for Bio SeNP extract_w (Table 3) may be caused by the intrinsic washing procedure, which could have removed a portion of β-strand containing proteins, as the former are more prone to interact with SeNPs directly [62]. Indeed, proteins featuring α-helix secondary structures have a higher degree of freedom than those containing β-strand motifs. This could potentially favor the adsorption of the former onto the SeNP surface and the consequent formation of a capping layer, as reported for cadmium selenide Quantum Dots [62]. Moreover, the β-sheet IR signal can, at least partially, derive from the formation of α-helix aggregates upon washing, whose IR contributions falls into the β-sheet spectral range [62]. Nevertheless, the higher thermodynamic stability observed for SeNPs within the Bio SeNP extract than the washed one (Figure 2 and Table 2) might suggest that β-strand secondary structures played a crucial role in avoiding NP aggregation or morphology transition, being in line with the identification of enzymes, such as alcohol dehydrogenases, which mostly contain orthogonally packed β-sheet motifs [63], as stabilizers for biogenic SeNPs [52]. This hypothesis was further supported by the high presence of β-strand structures in the OM (Table 3), which can be ascribed to the washing procedure used for its recovery that may have determined the stripping off of proteins featuring these motifs present in their larger content than α-helix ones (Table 3).

In addition, the spectral deconvolution featured IR signals typical of amide II band (ca. 1540 cm^−1^), as well as free amino acid residues (ca. 1595 and 1510 cm^−1^) (Figure S4 and Tables S5–S7). Similarly to the amide I band, that of amide II is sensitive to the secondary structure of proteins [64]; thus, a modification of this IR contribution may indicate variations occurring in protein motifs in response to SeO_3_^2−^ stress. In this regard, PCA performed for protein IR signals (97% of the original information) revealed how the samples were mainly discriminated along PC1 (47%), whose most relevant contributors were the amide II bands centered at ca. 1545 and 1560 cm^−1^ (i.e., amide II_I_ and II_II_) (Figure S3c,d). The latter, along with other amide II IR signals (1543 and 1522 cm^−1^), was fundamental for the projection of IR contributions deriving from *Micrococcus* sp. cells exposed for 24 h to SeO_3_^2−^ (Figure S3c and Table S6), likely indicating an active response of this environmental isolate to the presence of this oxyanion [65]. As opposed to this trend, Bio SeNP extract_w formed its own cluster (Figure S3c), due to the low value of amide II area (Table S7), further supporting the occurrence of important physical-chemical changes upon washing of the original extract. However, it is worth noting that amide II contribution is not considered a good predictor for the determination of protein motifs [64], as this vibrational mode can partially overlap, for instance, with COO^−^ asymmetric stretching vibration (ca. 1570 cm^−1^) typical of polysaccharides [30]. Indeed, amide I signals allowed for a finer discrimination of the samples based on PC2 (33%) and PC3 (17%), being the vectors referring to β-sheet and β-turn motifs linearly dependent (Figure S3d). Specifically, Bio SeNP extract and bacterial cells exposed for 120 h to the oxyanion clustered together (Figure S3c), as they featured comparable β-strand secondary structures likely deriving from the bacterial interaction with SeO_3_^2−^. The second discrete cluster grouped *Micrococcus* sp. cells incubated either in the absence (for 24 and 72 h) or the presence (72 h) of the oxyanion, which shared similar IR contributions referring to α-helix motifs (Table 3; Figure S3c and Figure S4; Tables S5 and S6), while unchallenged bacterial cells grown for 24 h stood alone (100% of α-helix motifs) (Figure S3c), highlighting the absence of any sort of stress (Figure S2a).

#### 3.3.3. Polysaccharides and Nucleic Acids

The presence of polysaccharides was indicated by the high IR absorption detected in the 1200–900 cm^−1^ region of the spectra (Figure 3), which was attributed to several -COH, -COC, -CC, -CO, -CH, -CH_2_, and -P=O vibrations (Tables S3 and S4). IR contributions typical of these biomolecules were better assigned through spectral deconvolutions of the region between 1550 and 950 cm^−1^ (Figure S5; Tables S8–S10). IR absorption bands of α_(1,3)_, α_(1,4)_, β_(1,3)_, and β_(1,4)_ glycosidic bonds were observed between 1200 and 950 cm^−1^ (Tables S8–S10) [66] and ascribed to the presence of peptidoglycan, teichoic, lipoteichoic, and teichuronic acids on the bacterial cell wall, which, being *Micrococcus* sp. a Gram-positive bacterium, can account for more than 60% of cell wall weight [67]. Since teichoic and lipoteichoic acids are carbohydrate polyols linked through phosphodiester bonds [67], the high-intensity IR signals typical of PO_2_^−^ groups (Tables S8–S10) further confirmed the detection of these biomolecules. Yet, absorption bands centered at ca. 1230 and 980 cm^−1^ were more likely attributable to phosphodiester functional groups of nucleic acids [68]. PCA performed on IR contributions typical of polysaccharides accounted for 88% of the original information and identified three PCs, where PC1, PC2, and PC3 represented the 51, 21, and 15% of variability, respectively (Figure S3e,f). In this case, PC1 separated the samples for IR signals attributed to β_(1,4)_ glycosidic bonds, -PO, and asymmetric -COO^−^ stretching vibrations, being the latter discriminant for the projection of unchallenged *Micrococcus* sp. cells actively grown for 24 h (Figure S3e,f). Moreover, the strong linear relationship between vectors representing β_(1,4)_ glycosidic bonds and -PO stretching vibrations typically occurring in the bacterial cell wall [69] is consistent with the large biomass yield detected (Figure S2a). α_(1,4)_ and β_(1,3)_ glycosidic bonds, as well as -CH2 scissoring and -C(OH) deformation modes, were instead relevant in the case of PC2 and PC3 (Figure S3f). Indeed, these PCs further differentiated the samples, highlighting the occurrence of three clusters that grouped (i) bacterial cells grown either in the absence (72 h) or presence (24 and 72 h) of SeO_3_^2−^, (ii) those after 120 h of SeO_3_^2−^ exposure and the recovered biogenic SeNP extract, likely due to the adsorption of polysaccharides onto the SeNP surface, and (iii) the OM with unchallenged cells incubated for 120 h (Figure S3e). The latter, alongside the clustering distance and the disappearance of several polysaccharide and nucleic acid IR vibrational modes observed in the Bio SeNP extract_w (Figure S3e and Figure S5i; Table S10; Table S10), indicated that partial removal of these biomolecules occurred during the washing procedure [30,31]. This phenomenon may suggest a less strong preference for these biomolecules to directly interact with SeNPs, whose removal however, determined a decrease in the surface charge (Table 2) and NP aggregation (Figure 2) of the Bio SeNP extract_w. Besides polysaccharides and nucleic acids, vibrational modes typical of fatty acids (1465–1440 cm^−1^), carboxylic acids (1415–1380 cm^−1^), ester moieties (1300–1290 cm^−1^), and amide III (ca. 1260 cm^−1^) were detected [30,40,45,60], being the latter more prominent for Se-containing samples and the OM (Tables S8–S10).

#### 3.3.4. Involvement of Thiol Chemistry

The contribution of RSH chemistry in SeO_3_^2−^ reduction by *Micrococcus* sp. was firstly indicated by the larger loss of RSH observed for oxyanion-incubating cultures than those unchallenged (Figure S2b), which did not show any modification of the RSH pool during growth. A drastic decrease in RSH was detected over time upon SeO_3_^2−^ exposure, being the highest after 72 h of growth (Figure S2b). Thus, it is reasonable to suggest that Painter-type reactions involving RSH chemistry played a key role in the biotransformation of the oxyanion in *Micrococcus* sp. cells, as previously reported for other microorganisms [9,14,17,25,45,46,52]. Consequently to these observations, a putative assignment of the highly convoluted IR signals in the fingerprint region (900–250 cm^−1^) (Figure 3) was proposed based on DFT calculations performed on Se_8_ units interacting with L-cysteine and its derivatives (Figure S1; Tables S1 and S2). Indeed, although some of the detected contributions were attributed to amino acid residues, fatty acid chains, nucleic acids, alkyl halides, and carboxylic acids [68,70] (Tables S3 and S4), their complete identification solely based on the available literature was often impaired, due to the specificity of the fingerprint region itself. As a result, ATR-FTIR spectra of *Micrococcus* sp. cells exposed to SeO_3_^2−^ as well as the biogenic extracts showed several IR absorption bands belonging to RSH- and disulfide (RSSR)-containing molecules, which were more represented in the latter (Tables S3 and S4), likely due to the occurrence of good interaction between the identified chemical species and the SeNP surface. Indeed, following the Painter-type mechanism, the RSH pool is continually regenerated by enzyme(s) responsible for the bioconversion of the unstable intermediate RSSeSR to counteract the stress exerted by SeO_3_^2−^ and the produced ROS [9]. Nevertheless, RSSR can still be present within the cells, depending on the intrinsic tolerance of the bacterial strain towards this oxyanion, time of SeO_3_^2−^ exposure, and bacterial growth conditions. 

ROS generation during SeO_3_^2−^ bioconversion can also play a crucial role in the modification of RSH-containing proteins. For instance, **^·^**O_2_^−^ produced during the first step of oxyanion bioreduction is transformed, by the enzyme superoxide dismutase (SOD), to H_2_O_2_, which, in turn, undergoes degradation processes mediated by catalase and peroxidase generating H_2_O [9]. However, an excess of H_2_O_2_ causes (i) the formation of RSSR bridges between diverse proteins, (ii) the attachment of low molecular thiols to the RSH moieties of proteins (i.e., S-thiolation), and (iii) the overoxidation of cysteine residues into cysteine sulfinic (RSO_2_H) and sulfonic (RSO_3_H) acids [71]. Hence, the loss of reduced RSH (Figure S2b) alongside the identification of RSSR-, RSO_2_H, and RSO_3_H vibrational modes in ATR-FTIR spectra of Se-containing samples (Tables S3 and S4) may derive also from the strong oxidative stress exerted by SeO_3_^2−^ in bacterial cells. Furthermore, DFT calculations highlighted a partial overlapping, in the 1370–1020 cm^−1^ region, between IR signals of polysaccharides and nucleic acids and those of RSH-containing or deriving compounds (Tables S1 and S2), which might elucidate the significant differences, in terms of peak position and area, observed between ATR-FTIR spectra of *Micrococcus* sp. cells either in the absence or presence of SeO_3_^2−^ (Tables S8 and S9). Indeed, IR bands related to the presence of RSH groups (e.g., 1150 and 1037 cm^−1^) decreased in their width and area from 72 h of the bacterial growth onwards under SeO_3_^2−^ conditioning, while these contributions were constant for oxyanion-free culture (Tables S8 and S9). Moreover, spectral deconvolutions featured IR peaks related to thiolate (RS^−^), sulfinate (RSO_2_^−^), sulfonate (RSO_3_^−^), RSO_2_H, RSO_3_H, RSSR, disulfide mono (RSOR), and dioxide (RSO_2_R) moieties (Tables S1, S2 and S9) either as free groups or adsorbed onto the SeNP surface only for *Micrococcus* sp. cells incubated in the presence of SeO_3_^2−^ and the biogenic extracts. These results were in line with the observed loss of RSH pool upon oxyanion bacterial incubation (Figure S2b), further suggesting the involvement of RSH buffering molecules in the microbial response to the stress exerted by SeO_3_^2−^.

**Figure 4:**
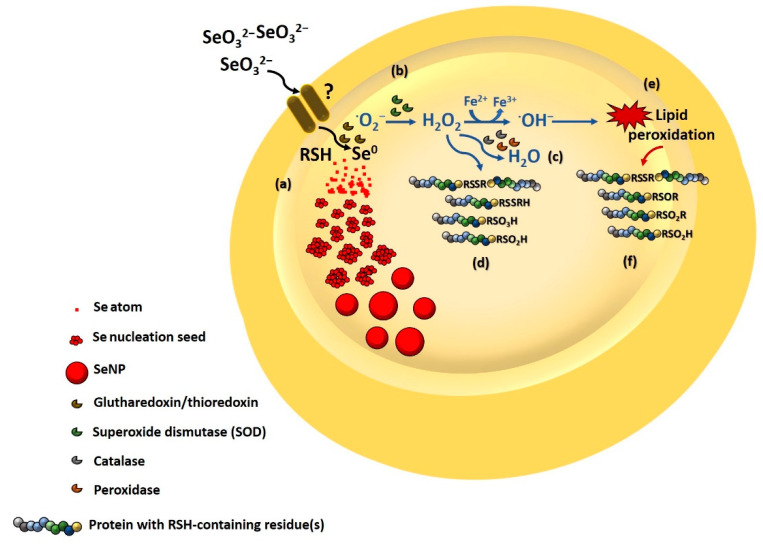
SeO_3_^2−^ is bioconverted into Se^0^ and **^·^**O_2_^−^ likely through Painter-type reaction involving intracellular RSH-containing molecules and enzymes with a high affinity for the RSSeSR intermediate, such as glutharedoxin and/or thioredoxin. This bioprocess causes a build-up of (**a**) Se atoms in the intracellular environment, which eventually aggregate with each other, and (**b**) **^·^**O_2_^−^ that are generally converted by superoxide dismutase (SOD) into H_2_O_2_. Once a certain solubility threshold of Se atoms is reached, Se-nucleation seeds are produced and aggregate forming SeNPs to counteract their thermodynamic instability, while (**c**) H_2_O_2_ is usually transformed by catalases and/or peroxidases into H_2_O. However, an excess of this ROS can lead to (**d**) modifications in RSH-containing proteins (i.e., disulfide bridges, S-thiolation, and overoxidation of cysteine residues to sulfinic and sulfonic acids) and (**e**) the peroxidation of polyunsaturated fatty acids of the bacterial membrane. The latter, in turn, can give rise to (**f**) further modifications of RSH-containing proteins and/or molecules, such as the generation of disulfide bridges, sulfinic acids, and cysteine mono- and di-oxide residues.

RSOSR and RSO_2_SR IR contributions (Tables S3, S4, S9 and S10) can arise from lipid peroxidation phenomena. Indeed, H_2_O_2_ can oxidize the Fe^2+^ pool through the Fenton reaction simultaneously determining the generation of ^·^OH^−^, which can cause lipid peroxidation of polyunsaturated fatty acids in the bacterial membrane [72]. The as-formed lipid peroxides can then interact with RSH-containing proteins in their proximity, resulting in the formation of RSSR-, RSO_2_H-, RSOR-, and RSO_2_R-moieties or residues [73]. Further evidence for this hypothesis can be indicated by the general and shared variations of -C=O stretching vibrations observed for Se-containing samples (Tables S6, S7, S9 and S10), reasonably caused by the generation of aldehydes, ketones, and carboxylic acids consequently to lipid peroxidation phenomena [74]. Indeed, ATR-FTIR spectra of *Micrococcus* sp. cells incubated with SeO_3_^2−^ revealed a general decrease in -CO stretching vibration (1740–1730 cm^−1^), due to the breaking of acyl-bonds in lipids, and the appearance of an IR absorption shoulder at ca. 1689 cm^−1^ attributable to aldehyde formation (Table S6) [75]. On the same line, the higher IR contributions in the 1420–1350 cm^−1^ region noticed for Se-containing samples than *Micrococcus* sp. SeO_3_^2−^-free culture (Tables S8–S10) can be due to the asymmetric -COO^−^ stretching vibrations deriving from carboxylic groups and the generation of α,β unsaturated aldehydes consequently to lipid peroxide modification [74,75]. Specifically, the largest IR absorption within this spectral region was observed for bacterial cells incubated for 72 h with oxyanion (Table S9), which agreed with the highest extent of RSH loss (Figure S2b), reasonably due to the strong oxidative stress resulting from SeO_3_^2−^ biotransformation for detoxification purposes [9]. Moreover, IR vibrational modes related to -PO_2_^−^ bands of phosphodiester bonds (ca. 1280–1200 cm^−1^) were subjected to significant changes, in terms of both intensity and peak position, within Se-containing samples (Tables S9 and S10), which might indicate a diverse structural organization of lipids caused by SeO_3_^2−^ bioprocessing [75]. A schematic representation tentatively summarizing SeO_3_^2−^ transformation, SeNP formation, and the consequent oxidative damage likely exerted onto *Micrococcus* sp. cells is proposed in Figure 4.

Overall, RSH-containing molecules and their derivatives were abundantly detected for bacterial cells exposed to SeO_3_^2−^ and the two biogenic SeNP extracts, while only a few IR signals typical of these functional groups were observed for the other samples, including the recovered OM (Tables S3–S10). Since most of the RSH modifications caused by SeO_3_^2−^ bioconversion likely occurred in low molecular weight thiols or proteins, it is reasonable to suggest that these biomolecules were more prone to adsorb onto the SeNP surface as compared to lipids, nucleic acids, and polysaccharides, therefore confirming the results obtained by analyzing the 1700–1500 cm^−1^ region of ATR-FTIR spectra (Tables S6 and S7). Besides, these modified proteins can contribute to the negative yet smaller surface charge of Bio SeNP extract_w than Bio SeNP extract (Table 2), as a portion of polysaccharides, lipids, and nucleic acids was removed from the latter upon washing.

#### 3.3.5. Biomolecule Contribution to SeNP Stability

**Figure 5:**
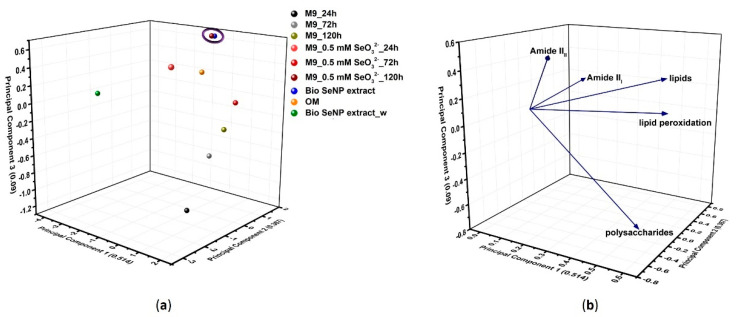
Representation of (**a**) score and (**b**) loading plots obtained through PCA performed on IR absorption bands contributing the most to samples’ variability (i.e., amide II, lipids, lipid peroxidation, and polysaccharides).

Based on the results presented above, IR absorption bands contributing the most to the variability of samples (i.e., those determining the separation for PC1 and referring to lipid peroxidation) were chosen to conduct a multivariate statistical analysis to obtain an overall picture regarding biomolecules involved in oxyanion bioprocessing, interaction, and stabilization of SeNPs. PCA performed on these specific IR contributions accounted for 97% of the original information, whose 51% was represented by PC1, while PC2 and PC3 related to 37 and 9% of variability (Figure 5).

**Figure 6:**
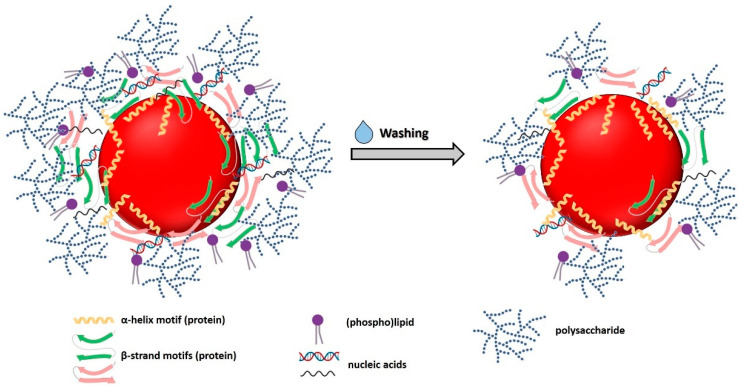
SeNPs produced by *Micrococcus* sp. cells are surrounded by an organic material that mediates their thermodynamic stabilization through electrosteric interactions. Proteins strongly interact with SeNPs, while lipids, polysaccharides, and nucleic acids appeared to be more loosely adsorbed onto the NP surface. Indeed, upon washing of the Bio SeNP extract, a depletion of these biomolecules is observed, alongside a decrease in β-strand protein motifs, suggesting that α-helix secondary structures may be more prone to interact with SeNPs, although they are not sufficient for maintaining NP stability.

PC1 separated the samples mostly based on the contributions deriving from polysaccharides, lipids, and lipid peroxidation, being the latter, most represented within *Micrococcus* sp. cells exposed for 72 h to SeO_3_^2−^ (Figure 5a), due to the oxidative damage exerted by this oxyanion. Similarly, bacterial cells grown for 120 h in the absence or presence of SeO_3_^2−^, those exposed for 24 h to the oxyanion, and Bio SeNP extract displayed significant lipids and lipid peroxidation IR vibrational modes (Figure 5) attributable to the supplying of either glucose or glucose and SeO_3_^2−^ as a stress source. The amide II bands were fundamental for PC2, highlighting the diversity of *Micrococcus* sp. cells incubated for 24 h with the oxyanion (Figure 5), which was the sample featuring the largest and most convoluted signal for this vibrational mode (Figure S3 and Table S6). This result indicated the occurrence of important protein modifications from the beginning of bacterial growth when SeO_3_^2−^ was added. In the case of PC3, most of the variability was attributed to the modified amide II and polysaccharide IR peaks (Figure 5b). The latter contribution resulted discriminant for ATR-FTIR spectra of Se-containing samples (localized in the positive quadrants) and those referring to *Micrococcus* sp. SeO_3_^2−^-free culture (negative quadrants) (Figure 5a), reflecting the higher growth extent observed when the oxyanion was absent (Figure S2a). Indeed, the largest polysaccharide bands were detected for unchallenged cells grown for 24 and 72 h (Figure 5a), which showed a higher biomass yield than other cell samples (Figure S2a). Only one cluster was observed performing PCA, which grouped *Micrococcus* sp. cells exposed for 120 h to SeO_3_^2−^ and the Bio SeNP extract (Figure 5a), whose similarity derived from SeNP extraction itself, which was carried out after 120 h of bacterial growth. The OM was relatively close to the Bio SeNP extract (Figure 5a), as the former was directly recovered from the biogenic extract. Finally, a shared feature of all identified PCs was the strong distance detected for the Bio SeNP extract_w (Figure 6), which related to the loss of polysaccharides, lipids, and, in a smaller portion, proteins, due to the washing procedure. A graphic representation displaying the complexity of the Bio SeNP extract and the adsorption of biomolecules, within the OM, onto the SeNP surface is proposed in Figure 6.

Based on the obtained results, the preferential adsorption of proteins onto the SeNP surface [52] can be linked to the occurrence of multiple interactions between NP and diverse functional groups of these biomolecules. Indeed, proteins may interact, through their headgroups, with the SeNP surface as both L-type and X-type ligands (two or one electron donors, respectively) [76]. Specifically, lone electron pair of -NH_2_ groups of proteins (L-type ligand) can coordinate with NP surface atoms, acting as a neutral donor, while an electron is needed to ensure an interaction between -COO^−^ or -RS^−^ moieties (X-type ligand) and NPs [76]. This phenomenon can result even more emphasized in the case of SeNPs produced by *Micrococcus* sp. cells, as several vibrational modes typical of -RS^−^ moieties, reasonably related to protein modifications caused by bacterial exposure to SeO_3_^2−^, were detected. Besides proteins, -COO^−^ and -OH groups of carbohydrates, as well as inorganic ions of nucleic acids, can act as X-type ligands onto the SeNP surface, contributing to the overall stabilization of these NMs [76]. Nevertheless, these biomolecules seemed to loosely interact with biogenic SeNPs, as their partial desorption or dissolution was observed upon washing of the Bio SeNP extract, causing NP aggregation and morphology variation.

## 4. Conclusions

The metal-tolerant *Micrococcus* sp. showed its proficiency, under metabolically controlled growth conditions, of bioconverting SeO_3_^2−^ into Se^0^, likely through Painter-type reaction involving RSH-containing molecules, producing spherical and thermodynamically stable SeNPs with an average diameter of 149 nm. The stability of these SeNPs was mediated by the presence of an organic material surrounding them, which, based on ATR-FTIR spectroscopy, contained lipids, proteins, polysaccharides, and nucleic acids likely adsorbed onto the NP surface. Out of these biomolecules, proteins appeared to interact the most with SeNPs, as polysaccharides, lipids, and nucleic acids were substantially removed upon washing the obtained biogenic SeNP extract with water. These results, alongside the detection of big SeNPs, NP aggregates, SeNRs, and a decreased surface charge for Bio SeNP extract_w, indicated that the OM integrity was of paramount importance to maintain their thermodynamic stability through the occurrence of electrosteric interactions. In this regard, although proteins featuring α-helix motifs strongly interacted with SeNPs, their presence within the Bio SeNP extract_w was not sufficient to prevent NP aggregation or modification, likely due to the lower steric hindrance exerted by these biomolecules than β-strand proteins, lipids, and polysaccharides. Finally, new insights regarding the modification of RSH-containing molecules (i.e., low molecular weight thiols and proteins), consequently to SeO_3_^2−^ bioprocessing and oxidative stress, and their participation in SeNP stabilization were obtained through DFT calculations.

## Figures and Tables

**Table 1:** Nomenclature and description of the investigated samples.

Samples	Description
M9_24 h	Aliquot of 24 h-unchallenged *Micrococcus* sp. culture
M9_72 h	Aliquot of 72 h-unchallenged *Micrococcus* sp. culture
M9_120 h	Aliquot of 120 h-unchallenged *Micrococcus* sp. culture
M9_0.5 mM SeO_3_^2−^_24 h	Aliquot of *Micrococcus* sp. culture exposed for 24 h to SeO_3_^2−^
M9_0.5 mM SeO_3_^2−^_72 h	Aliquot of *Micrococcus* sp. culture exposed for 72 h to SeO_3_^2−^
M9_0.5 mM SeO_3_^2−^_120 h	Aliquot of *Micrococcus* sp. culture exposed for 120 h to SeO_3_^2−^
Bio SeNP extract	Biogenic SeNP extract recovered from *Micrococcus* sp. cells incubated for 120 h with SeO_3_^2−^
Bio SeNP extract_w	Biogenic SeNP extract washed in 1 mL of ddH_2_O
OM	Organic material recovered from biogenic SeNP extract

**Table 2:** Surface charges of biogenic suspensions.

Sample	ζ Potential Value (mV)
Bio SeNP extract	−27.2 ± 0.7
Bio SeNP extract_w	−21.1 ± 0.4
OM	−26.4 ± 0.5

**Table 3:** Estimation of diverse protein conformations based on amide I component analysis.

Samples	A_amide I_	β-Antiparallel (%)	β-Turn (%)	α-Helix (%)	β-Sheet (%)
M9_24 h	40.86	-	-	100	-
M9_72 h	38.99	-	1	99	-
M9_120 h	39.80	-	1	99	-
M9_0.5 mM SeO_3_^2−^_24 h	55.63	-	25	19	56
M9_0.5 mM SeO_3_^2−^_72 h	42.11	2	-	98	-
M9_0.5 mM SeO_3_^2−^_120 h	50.14	-	35	24	41
Bio SeNP extract	49.79	-	41	19	40
OM	62.68	8	-	28	64
Bio SeNP extract_w	46.36	-	-	79	21

“-“ indicates the absence of the specific IR contribution in the spectral deconvolution for the amide I region.

## Data Availability

Not applicable.

## Supplementary Materials

The following are available online at https://www.mdpi.com/article/10.3390/nano11051195/s1, Figure S1: Thiol (RSH)-containing and deriving species on which Density Functional Theory (DFT) calculations were performed, either as isolated molecules or interacting with a Se_8_ unit, where R-S^−^, R-SO_2_H, R-SO_2_^−^, R-SO_3_H, R-SO_3_^−^, R-S-S-R, R-SO-S-R, and R-SO_2_-S-R represent thiolate, sulfinic acid, sulfinate, sulfonic acid, sulfonate, disulfide, disulfide monoxide, and disulfide dioxide moieties, respectively, Table S1: Density Functional Theory (DFT) calculations for IR vibrations performed considering free thiol-containing molecules (i.e., L-cysteine and its derivatives) and their adsorption on Se_8_ nuclei, Table S2: Density Functional Theory (DFT) calculations for IR vibrations performed considering cystine and its derivatives and their adsorption on Se_8_ nuclei, Figure S2: (a) Growth profile and (b) RSH depletion of Micrococcus sp. cells in M9 medium supplied with either glucose or glucose and SeO_3_^2−^, Table S3: ATR-FTIR absorption bands and identification of Micrococcus sp. unchallenged cells or exposed to SeO_3_^2−^, Table S4: ATR-FTIR absorption bands and identification of biogenic samples (Bio SeNP extract, OM, and Bio SeNP extract_w), Figure S3: Representation of (a,c,e) score and (b,d,f) loading plots obtained by performing PCA on IR contributions referring to (a–b) lipids, (c–d) proteins, and (e–f) polysaccharides of Micrococcus sp. cells incubated in the presence/absence of SeO_3_^2−^ and the derived Bio SeNP extracts, alongside OM, Figure S4: ATR-FTIR spectral deconvolution performed on the 1780–1480 cm^−1^ region for Micrococcus sp. cells incubated for (a,d) 24 h, (b,e) 72 h, and (c,f) 120 h with either (a–c) glucose or (d–f) glucose and SeO_3_^2−^, (g) Bio SeNP extract, (h) OM, and (i) Bio SeNP extract_w, Table S5: Deconvolution of ATR-FTIR spectra of Micrococcus sp. unchallenged cells in the 1780–1480 cm^−1^ region, Table S6: Deconvolution of ATR-FTIR spectra of Micrococcus sp. cells incubated with SeO_3_^2−^ in the 1780–1480 cm^−1^ region, Table S7: Deconvolution of ATR-FTIR spectra of biogenic SeNP extracts and OM in the 1780–1480 cm^−1^ region, Figure S5: ATR-FTIR spectral deconvolution performed on the 1500–800 cm^−1^ region for Micrococcus sp. cells incubated for (a,d) 24 h, (b,e) 72 h, and (c,f) 120 h with either (a–c) glucose or (d–f) glucose and SeO_3_^2−^, (g) Bio SeNP extract, (h) OM, and (i) Bio SeNP extract_w, Table S8: Deconvolution of ATR-FTIR spectra of Micrococcus sp. unchallenged cells in the 1500–800 cm^−1^ region, Table S9: Deconvolution of ATR-FTIR spectra of Micrococcus sp. cells incubated with SeO_3_^2−^ in the 1500–800 cm^−1^ region, Table S10: Deconvolution of ATR-FTIR spectra of biogenic SeNP extracts and OM in the 1500–950 cm^−1^ region.
