# Supplementary material for: Biogenic Selenium Nanoparticles: A Fine Characterization to Unveil Their Thermodynamic Stability
Source: Nanomaterials (Basel). 2021 May 1;11(5):1195. doi: 10.3390/nano11051195 (PMC8147324; doi:10.3390/nano11051195)
Supplement: Supplementary file 1 [file nanomaterials-11-01195-s001.zip › nanomaterials-1201566-supplementary-conversion.pdf]

nanomaterials

MDPI

*Supplementary material for Article*

# Biogenic Selenium Nanoparticles: A Fine Characterization to Unveil Their Thermodynamic Stability

**Elena Piacenza [1,*], Alessandro Presentato [1,*], Francesco Ferrante [2], Giuseppe Cavallaro [2], Rosa Alduina [1] and Delia F. Chillura Martino [1]**

[1] Department of Biological, Chemical, and Pharmaceutical Sciences and Technologies (STEBICEF), University of Palermo, Viale delle Scienze Ed. 16, 90128, Palermo, Italy; elena.piacenza91@gmail.com; alessandro.presentato@unipa.it; valeria.alduina@unipa.it; delia.chilluramartino@unipa.it

[2] Department of Physics and Chemistry "Emilio Segrè" (DIFC), University of Palermo, Viale delle Scienze Ed. 17, 90128, Palermo, Italy; francesco.ferrante@unipa.it; giuseppe.cavallaro@unipa.it

* Correspondence: elena.piacenza91@gmail.com; alessandro.presentato@unipa.it

**Citation:** Piacenza, E.; Presentato, A.; Ferrante, F.; Cavallaro, G.; Alduina, R.; Chillura Martino, D.F. Biogenic Selenium Nanoparticles: A Fine Characterization to Unveil Their Thermodynamic Stability. *Nanomaterials* **2021**, *11*, 1195. https://doi.org/10.3390/nano11051195

Academic Editor: Andrés Guerrero-Martínez

Received: 13 April 2021
Accepted: 29 April 2021
Published: 1 May 2021

**Publisher's Note:** MDPI stays neutral with regard to jurisdictional claims in published maps and institutional affiliations.

**Copyright:** © 2021 by the authors. Submitted for possible open access publication under the terms and conditions of the Creative Commons Attribution (CC BY) license (http://creativecommons.org/licenses/by/4.0/).

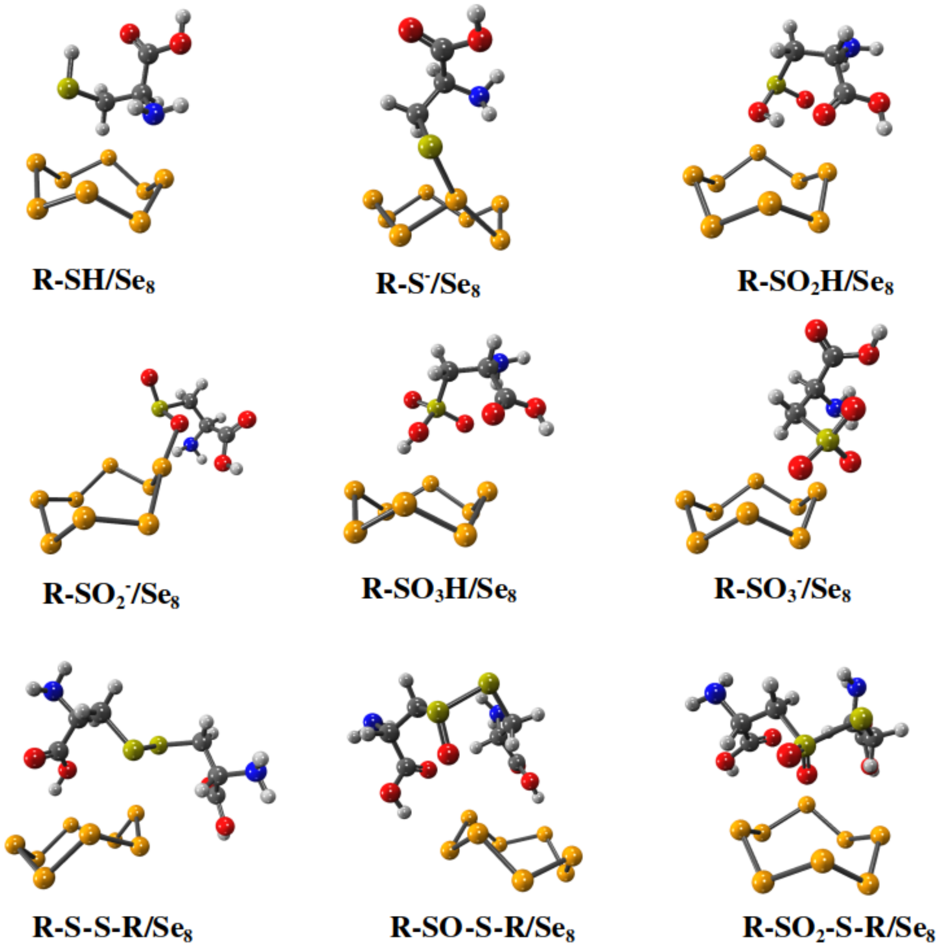


**Figure S1:** Thiol (RSH)-containing and deriving species on which Density Functional Theory (DFT) calculations were performed, either as isolated molecules or interacting with a $Se_8$ unit, where R-$S^-$, R-$SO_2$H, R-$SO_2^-$, R-$SO_3$H, R-$SO_3^-$, R-S-S-R, R-SO-S-R, and R-$SO_2$-S-R represent thiolate, sulfinic acid, sulfinate, sulfonic acid, sulfonate, disulfide, disulfide monoxide, and disulfide dioxide moieties, respectively.

**Table S1.** Density Functional Theory (DFT) calculations for IR vibrations performed considering free RSH-containing molecules (*i.e.*, L-cysteine and its derivatives) and their adsorption on $Se_8$ nuclei.

| Vibrational modes | $\tilde{\nu}$ ($cm^{-1}$) | | | | | | | | | | | |
|---|---|---|---|---|---|---|---|---|---|---|---|---|
| | R-SH | R-SH/$Se_8$ | R-$S^-$ | R-$S^-$/$Se_8$ | R-$SO_2$H | R-$SO_2$H/$Se_8$ | R-$SO_2^-$ | R-$SO_2^-$/$Se_8$ | R-$SO_3$H | R-$SO_3$H/$Se_8$ | R-$SO_3^-$ | R-$SO_3^-$/$Se_8$ |
| ν (S)OH | | | | | 3449 | 3485 | | | | 3555 | | |
| ν ($NH_2$) | 3456 | 3456 | 3495 | 3534 | 3543 | 3545 | 3485 | 3515 | 3547 | 3548 | 3521 | 3533 |
| | | | 3253 | 3447 | 3462 | 3461 | 3379 | 3446 | 3473 | 3473 | 3240 | 3423 |
| ν ($CH_2$) | 3149 | 3161 | 3011 | 3119 | 3148 | 3149 | 3127 | 3137 | 3183 | 3182 | 3140 | 3112 |
| | 3086 | 3099 | 2950 | 3058 | 3077 | 3077 | 3036 | 3059 | 3102 | | 3068 | 3081 |
| ν (CH) | 2986 | 2984 | 2927 | 2988 | 2997 | 2994 | 2949 | 2993 | 3015 | 3012 | 2952 | 2991 |
| ν (CO) | 1816 | 1819 | 1801 | 1817 | 1780 | 1778 | 1805 | 1823 | 1831 | 1819 | 1814 | 1821 |
| δ ($NH_2$) | 1664 | 1638 | 1635 | 1646 | 1654 | 1652 | 1665 | 1667 | 1669 | 1662 | 1672 | 1655 |
| | 958 | 962 | 1270 | 1151 | 963 | 961 | 1124 | 987 | 947 | 953 | 1400 | 1398 |
| | 934 | 933 | 1014 | 973 | 400 | | 1021 | | 892 | | 1230 | 994 |
| | | | | 950 | | | 788 | | | | 1002 | 965 |
| | | | | 880 | | | | | | | 971 | |
| δ ($CH_2$) | 1423 | 1423 | 1430 | 1419 | 1423 | 1421 | 1419 | 1406 | 1410 | 1409 | 1412 | 1417 |
| | 1297 | 1300 | 1139 | 1286 | 1267 | 1270 | 1247 | 1263 | 1301 | 1307 | 1290 | 1301 |
| | 1257 | 1257 | 1121 | 1233 | 1238 | 1153 | 1203 | 1216 | 1236 | 1284 | 1191 | 1240 |
| | 1164 | 1163 | 892 | 1132 | 1130 | 1131 | 1106 | 1135 | 1142 | 1234 | 1167 | 1126 |
| | | 1142 | 814 | 809 | 992 | 992 | 955 | 1115 | 1007 | 1175 | 1108 | 1067 |
| | | | | | 864 | 865 | 851 | 967 | 823 | 1007 | 892 | 897 |
| | | | | | 853 | 833 | | 881 | | 823 | 827 | 827 |
| | | | | | | | | 819 | | | | |
| δ (CH) | 1389 | 1389 | 1322 | 1374 | 1396 | 1395 | 1390 | 1375 | 1394 | 1392 | 1332 | 1321 |
| | | | | | | 1303 | 1298 | 1312 | | 1292 | 1137 | 1169 |
| | | | | | | | | 456 | | | | |
| δ (OH) | 1373 | 1373 | 1349 | 1358 | 1391 | 1389 | 1354 | 1361 | 1378 | 1376 | 1363 | 1367 |
| | 1309 | 1308 | 1204 | 1183 | 1188 | 1189 | 1161 | 1184 | 1187 | 1188 | 1187 | 1194 |
| | 1179 | 1175 | 1178 | 747 | 755 | 755 | 735 | 756 | 753 | 752 | 758 | 1177 |
| | 741 | 743 | 742 | 675 | 663 | 671 | 609 | 647 | 685 | 676 | 577 | 760 |

| | | | | | | | | | | | | |
|---|---|---|---|---|---|---|---|---|---|---|---|---|
| | 668 | 670 | 670 | 632 | | 592 | 435 | 561 | 636 | 630 | 520 | 626 |
| | 634 | 633 | 613 | 560 | | | | 516 | | | 516 | 579 |
| | 551 | 554 | | | | | | 433 | | | | 535 |
| | | 546 | | | | | | | | | | |
| δ (S)OH | | | | | 1273 | 1249 | | | 1317 | 1144 | | |
| | | | | | 691 | 1221 | | | 1175 | 1132 | | |
| | | | | | | 639 | | | 1152 | 1083 | | |
| | | | | | | 578 | | | 1091 | | | |
| ν (SO) | | | | | 1063 | 1050 | 1047 | 1074 | 756 | 759 | 953 | 939 |
| | | | | | | | 915 | 792 | | | | |
| ν SO(H) | | | | | 733 | 730 | | | | | | |
| ν (CS) | | | | | | | | | | | 684 | 692 |
| δ (SH) | 1040 | 1038 | | | | | | | | | | |
| | 851 | 852 | | | | | | | | | | |
| | 779 | 777 | | | | | | | | | | |
| | 371 | 370 | | | | | | | | | | |
| | 334 | 337 | | | | | | | | | | |
| | | 315 | | | | | | | | | | |
| | | 274 | | | | | | | | | | |
| δ ($NH_2$) | 305 | | 373 | 270 | 356 | 355 | 421 | 321 | 360 | 360 | 350 | 361 |
| | | | | | | | | 296 | 319 | 328 | | |
| | | | | | | 322 | | 278 | | | | |
| backbone | 265 | 418 | 325 | 522 | 534 | 533 | 560 | 572 | 580 | 576 | 474 | 517 |
| | | | 277 | 443 | 455 | 456 | 518 | 377 | 522 | 523 | 409 | 476 |
| | | | | 346 | 415 | 415 | 514 | 262 | 506 | 504 | 357 | 369 |
| | | | | 305 | 324 | 390 | 462 | | 451 | 453 | 299 | 302 |
| | | | | 258 | 292 | | 318 | | 416 | 425 | 269 | |
| | | | | | | | 261 | | 397 | 418 | | |
| | | | | | | | | | 293 | 268 | | |
| δ ($SO_3$) | | | | | | | | | | | 468 | 474 |
| δ OH ($HSO_3$) | | | | | | | | | 282 | 341 | | |
| $Se_8$ | | 262-257 | | 281-251 | | 264-258 | | 266-251 | | 265-253 | | |

Where ν and δ indicate stretching and bending vibrations.

**Table S2.** Density Functional Theory (DFT) calculations for IR vibrations performed considering cystine (RSSR) and its derivatives and their adsorption on $Se_8$ nuclei.

| Vibrational modes | $\tilde{\nu}$ (cm$^{-1}$) | | | | | |
|---|---|---|---|---|---|---|
| | R-S-S-R | R-S-S-R/$Se_8$ | R-SO-S-R | R-SO-S-R/$Se_8$ | R-$SO_2$-S-R | R-$SO_2$-S-R/$Se_8$ |
| ν ($NH_2$) | 3540 | 3540 | 3566 | 3570 | 3573 | 3573 |
| | 3538 | 3536 | 3518 | 3523 | 3533 | 3535 |
| | 3460 | 3459 | 3481 | 3482 | 3486 | 3485 |
| | 3454 | 3454 | 3444 | 3444 | 3450 | 3451 |
| ν ($CH_2$) | 3124 | 3132 | 3141 | 3147 | 3166 | 3166 |
| | 3103 | 3109 | 3111 | 3144 | 3162 | 3161 |
| | 3061 | 3065 | 3059 | 3073 | 3084 | 3088 |
| | 3034 | 3038 | 3022 | 3068 | 3068 | 3067 |
| ν (CH) | 3088 | 3084 | 3069 | 3071 | 3091 | 3074 |
| | 2965 | 2968 | 2914 | 2983 | 2987 | 2980 |
| ν (CO) | 1845 | 1842 | 1835 | 1814 | 1837 | 1838 |
| | 1831 | 1816 | 1834 | 1781 | 1812 | 1812 |
| δ ($NH_2$) | 1642 | 1642 | 1655 | 1647 | 1645 | 1647 |
| | 1627 | 1627 | 1617 | 1610 | 1614 | 1614 |
| | 951 | 953 | 903 | 1257 | 1267 | 1408 |
| | 883 | 880 | 863 | 951 | 1020 | 1266 |
| | | | | 926 | 921 | 1173 |
| | | | | 897 | 884 | 1020 |
| | | | | 862 | 855 | 921 |
| | | | | | | 883 |
| | | | | | | 852 |
| δ ($CH_2$) | 1420 | 1416 | 1433 | 1432 | 1432 | 1432 |
| | 1414 | 1405 | 1408 | 1422 | 1421 | 1422 |
| | 1244 | 1253 | 1265 | 140 | 1406 | 1259 |
| | 1110 | 1242 | 1198 | 1400 | 1256 | 1208 |
| | 1099 | 1225 | 1191 | 1140 | 1136 | 1194 |
| | 787 | 1192 | 1145 | 1133 | 1130 | 1110 |
| | 768 | 936 | 1007 | 1005 | 1106 | 1007 |
| | | 895 | 837 | 813 | 1007 | 961 |
| | | 629 | 761 | 638 | 958 | 828 |
| | | 622 | | | 826 | 822 |
| | | 582 | | | 819 | |
| δ (CH) | 1436 | 1459 | 1425 | 1371 | 1420 | 1405 |
| | 1370 | 1441 | 1359 | 1322 | 1370 | 1401 |
| | 1356 | 1374 | 1248 | 1276 | 1285 | 1375 |
| | 1325 | 1362 | 1136 | 1191 | 1207 | 1290 |
| | 1254 | 1149 | 1020 | 1189 | 1195 | |
| | 1224 | 1112 | 953 | 1108 | | |
| | 1148 | 1097 | 921 | 1017 | | |
| δ (OH) | 1321 | 1328 | 1398 | 1437 | 1343 | 1331 |
| | 1263 | 1319 | 1381 | 1352 | 1312 | 1314 |
| | 1190 | 1262 | 1335 | 1285 | 1273 | 1273 |
| | 1169 | 1174 | 1306 | 1210 | 1185 | 1175 |
| | 801 | 1145 | 1273 | 1204 | 1176 | 1141 |
| | 640 | 802 | 1178 | 830 | 771 | 1126 |

| | | | | | | |
|---|---|---|---|---|---|---|
| | 626 | 788 | 1164 | 780 | 754 | 758 |
| | 622 | 770 | 1120 | 758 | 681 | 749 |
| | 576 | 756 | 803 | 720 | 644 | 679 |
| | | 682 | 756 | 666 | 636 | 639 |
| | | 496 | 690 | 591 | 615 | 635 |
| | | | 648 | 506 | 581 | 591 |
| | | | 636 | 480 | 569 | 575 |
| | | | 594 | | | 569 |
| | | | 569 | | | 511 |
| | | | 486 | | | |
| | | | 483 | | | |
| ν (SO) | | | 1049 | 1037 | 1068 | 1069 |
| ν (CS) | 710 | 715 | 704 | 712 | 725 | 720 |
| | 708 | 710 | | 694 | | |
| | 397 | | | | | |
| | 370 | | | | | |
| backbone | 1049 | 1046 | | | 509 | 481 |
| | 1034 | 1029 | | | 482 | |
| | 936 | | | | | |
| | 896 | | | | | |
| | 755 | | | | | |
| | 710 | | | | | |
| | 503 | | | | | |
| | 287 | | | | | |
| ν (SS) | 485 | 487 | | | | |
| | 476 | 480 | | | | |
| δ ($NH_2$) | 356 | 404 | 342 | 418 | 352 | 354 |
| | 294 | 337 | 328 | 338 | 336 | 336 |
| | 274 | 317 | 316 | 325 | 313 | 319 |
| | 270 | 279 | 295 | 285 | 306 | 295 |
| | 259 | 269 | 282 | 270 | 293 | 278 |
| | | 268 | 265 | 263 | 273 | 257 |
| | | | | 256 | 251 | 254 |
| δ ($CH_2$) | 321 | 317 | 449 | 412 | 421 | 421 |
| | | 288 | 421 | 352 | 382 | 384 |
| | | | 391 | 303 | | |
| δ (SO) | | | 251 | 450 | | |
| δ ($SO_2$) | | | | | 467 | 470 |
| $Se_8$ | | 259-258 | | 263-254 | | 263 |

Where ν and δ indicate stretching and bending vibrations.

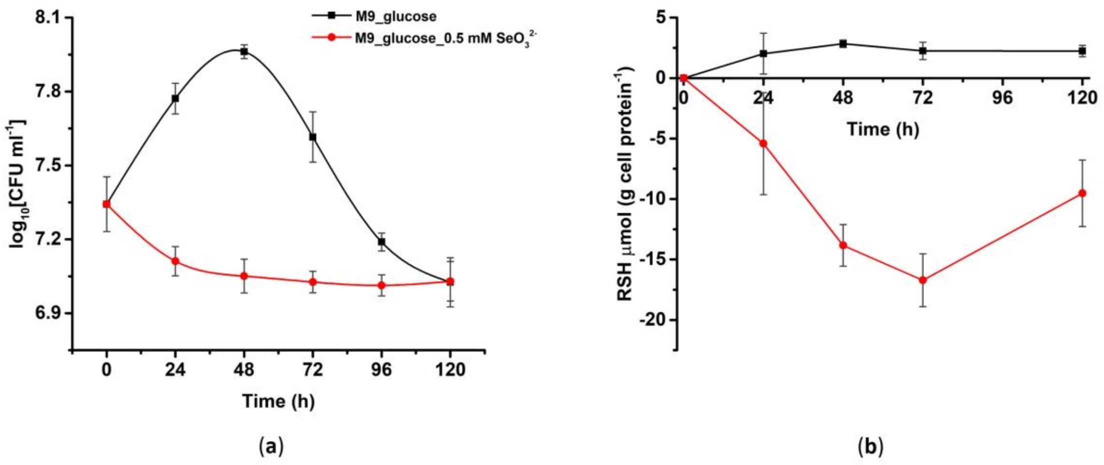


**Figure S2:** (**a**) Growth profile and (**b**) RSH depletion of *Micrococcus* sp. cells in M9 medium supplied with either glucose or glucose and $SeO_3^{2-}$

**Table S3.** ATR-FTIR absorption bands and identification of *Micrococcus* sp. unchallenged cells or exposed to $SeO_3^{2-}$.

| $\tilde{\nu}$ (cm$^{-1}$) | | | | | | Vibrational modes | Identification |
|---|---|---|---|---|---|---|---|
| **M9_24h** | **M9_72h** | **M9_120h** | **M9_0.5 mM $SeO_3^{2-}$_24h** | **M9_0.5 mM $SeO_3^{2-}$_72h** | **M9_0.5 mM $SeO_3^{2-}$_120h** | | |
| 3286 | 3284 | 3286 | 3285 | 3282 | 3284 | ν (NH) | Proteins (Amide A) [1] |
| 3070 | 3068 | 3068 | 3069 | 3062 | 3067 | $\nu_s$ ($NH^{3+}$) | Proteins (Amide B) [2] |
| 2955 | 2953 | 2962 | 2960 | 2958 | 2962 | $\nu_{as}$ ($CH_3$) | Fatty acids [1-3] |
| 2923 | 2921 | 2924 | 2925 | 2926 | 2928 | $\nu_{as}$ ($CH_2$) | Fatty acids [1-3] |
| 2869 | 2876 | 2872 | 2875 | 2872 | 2873 | $\nu_s$ (CH) | Amino acids in fatty acids [3] |
| 2856 | 2850 | 2852 | 2854 | 2853 | 2855 | $\nu_s$ ($CH_2$) | Fatty acids [1-3] |
| 1742 | 1739 | 1740 | 1736 | 1740 | 1739 | ν (CO) | Ester moieties of lipids and polysaccha- |

| | | | | | | | |
|---|---|---|---|---|---|---|---|
| | | | | | | | rides [1-3] |
| 1652 | 1650 | 1651(s) | | | | $\nu$ (CO) | $\alpha$-helix proteins (Amide I) [1-3] |
| 1642(s) | 1643(s) | 1643 | 1646 | 1644 | 1645 | $\nu_{as}$ (COO$^-$); $\beta$ (NH$_2$); $\delta$ (NH) | $\beta$-sheet proteins [1] |
| | | | 1559(s) | 1561(s) | 1558(s) | $\delta$ (NH); $\nu$ (CN) | $\alpha$-helix proteins (Amide II) [1-3] |
| 1545 | 1544 | 1539 | 1541 | 1542 | 1539 | $\delta$ (NH); $\nu$ (CN) | $\alpha$-helix proteins (Amide II) [1-3] |
| | | | 1515(s) | 1514(s) | 1515(s) | $\delta_s$ (NH$_3^+$); $\delta$ (NH); $\nu$ (CN) | Amino acid residues [2]; RSH-containing molecules |
| 1467(s) | 1469(s) | 1468(s) | 1467(s) | 1468(s) | 1468(s) | $\delta$ (CH$_2$); $\delta$ (CH$_3$); $\beta$ (CH$_2$) | Lipids and proteins [3] |
| 1450 | 1452 | 1450 | 1448 | 1450 | 1451 | $\delta_{sciss}$ (CH$_2$); $\delta$ (OH); $\nu$ CC(O); $\nu_s$ (COO$^-$) | Polysaccharides [4]; aliphatic groups of proteins [5]; R-SO$_2$H-containing molecules adsorbed on SeNP |
| 1395 | 1394 | 1393 | 1395 | 1394 | 1395 | $\nu_s$ (COO$^-$) | Amino acid side chains; free fatty acids [3]; peptides [5] |
| 1385(s) | 1384(s) | 1385(s) | | | | $\delta$ (CH); $\delta$ (COH); $\beta$ (CH$_3$); $\nu$ CC(O); $\nu_s$ (COO$^-$); $\delta$ (NH$_2$); $\nu$ (CN) | Aldehydes; carboxylic acids; peptides [5]; aromatic amines [6] |
| | | | 1373(s) | 1377(s) | 1373(s) | $\beta$ (CH$_3$); $\delta$ (CH); $\delta$ (OH); | Lipids and proteins [3]; RSH-, RSO$_3$H-, RSSR-, and RSO$_2$SR-containing molecules; RSH-, RS$^-$-, RSO$_2^-$-, RSO$_3$H-, RSSR-, RSOSR-, and RSO$_2$SR-containing molecules adsorbed on SeNP |
| 1301 | 1299 | 1299 | 1298 | 1300 | 1300 | $\omega$ (CH$_2$); $\nu$ (CN); $\delta$ (NH$_2$); $\varrho_{as(oph)}$ (CH); $\nu$ (COC); $\nu$ (CCO); $\delta$ (OH); $\delta$ (CH) | Ester moieties [1]; proteins [3]; RSH-, RSO$_2^-$-, RSO$_3$H-, RSSR-, RSOSR-containing molecules; RSH-and RSO$_2$H-, RSO$_3$H-, RSO$_3^-$-containing molecules adsorbed on SeNP; |
| 1249 | 1246 | 1243 | 1244 | 1243 | 1243 | $\nu$ (CN); $\delta$ (NH$_2$) | Proteins (Amide III) [1] |
| 1149 | 1146 | 1147 | 1149 | 1150 | 1151 | $\nu_{as}$ (COC); $\delta$ (CH$_2$); $\delta$ (CH); $\delta$ (NH$_2$); $\delta$ (OH); $\delta$ (S)OH; $\nu$ (CO) | Nucleic acids; $\alpha_{(1,4)}$ glycosidic bonds [7]; polysaccharide ring [8]; amino acids [9]; RSH-containing molecules |

| | | | | | | | |
|---|---|---|---|---|---|---|---|
| 1102(s) | 1099(s) | 1101(s) | 1099(s) | 1102(s) | 1101(s) | $\nu_{as}$ (COC); ν (CC); ν (CO); δ (COH); ν $P(OH)_2$ | $\beta_{(1,4)}$ glycosidic bonds [7]; amino acids [9]; polysaccharides [4,8] |
| 1075 | 1076 | 1070(s) | 1073 | 1074(s) | 1071(s) | ν (CO); ν (CC); ν (COH); δ (COC); ϱ ($NH_3^+$); ν (SO) | Polysaccharides, proteins, and polyesters [10]; amino acid residues [11]; $RSO_2^-$-containing molecules adsorbed on SeNP |
| 1062 | 1063 | | | | | ν (CO); ϱ (CO); $\nu_s$ ($PO_2^-$); ν (CC); ν (C)OH; ν (COH); δ (COC) | Nucleic acids [4]; phospholipids [4]; $\beta_{(1,3)}$ glycosidic bonds [7]; polysaccharides [1] |
| 1049(s) | 1050(s) | | 1055 | 1054 | 1055 | ν (CC); ν (CO); δ (COH); ν (SO) | Polysaccharides [9]; $RSO_2H$-containing molecules adsorbed on SeNP |
| 1037 | 1037 | 1035 | 1038 | 1036 | 1038 | ν (PO); ν (SH); ν (SO); backbone vibration | Polysaccharides and nucleic acids [1-4]; RSSR-containing molecules; RSH- and RSOSR-containing molecules adsorbed on SeNP |
| 990 | 988 | 985(s) | 990 | 982(s) | 982(s) | δ ($NH_2$); δ (HNCC); ν (CO); $\nu_s$ ($PO_3^{2-}$); δ (COH); ν (CC) | Amino acids [9,12]; $\beta_{(1,3)}$ glycosidic bonds [7]; nucleic acids [11]; polysaccharides [8]; $RS^-$-containing molecules adsorbed on SeNP |
| 922 | 921 | 922 | 920 | 921 | 922 | δ (=CH); τ ($CH_2$); $\nu_s$ ($PO_4^{3-}$); δ (SH); ν (SO); δ ($NH_2$); δ ($CH_2$) | Alkyl halides, carboxylic acids; amines; $\alpha_{(1,3)}$ glycosidic bonds [7]; amino acid residues [9]; nucleic acids [4]; RSH-, RSSR-, $RSO_2SR$-containing molecules; RSSR-, RSOSR-, $RSO_2SR$-containing molecules adsorbed on SeNP |
| 860 | 857 | 860 | 863 | 862 | 863 | δ ($NH_2$); ν (CC); ν (CN) | Amino acid residues [9]; $RSO_2H$-containing molecules adsorbed on SeNP |
| 802 | 803 | 802 | 802 | 800(s) | 796(s) | ν SO(H); δ (OH) $\delta_{op}$ (HOCC); ν (PO) | Amino acids [9]; nucleic acids [13]; RSH-, $RS^-$-, RSSR-containing molecules; $RS^-$-, RSOSR-containing molecules ad- |

| | | | | | | | |
|---|---|---|---|---|---|---|---|
| | | | | | | | sorbed on SeNP |
| 779 | 779 | 779 | 778 | 777 | 777 | ν C(COOH); δ ($NH_2$); ϱ ($CH_2$); δ (HNC); δ (CCH); δ (OH) | Amino acids [9]; RSH-, RSSR-, RSOSR-, $RSO_2SR$-containing molecules; $RSO_3^-$-containing molecules adsorbed on SeNP |
| | | | | 754 | 755 | | |
| | | | 739 | 738 | 738 | δ ($NH_2$)C(COOH); ϱ ($CH_2$); δ (OH) | Amino acids [9]; RSH-, $RS^-$-, $RSO_2H$-, $RSO_2^-$-, $RSO_2SR$-containing molecules adsorbed on SeNP |
| 722 | 724 | 723 | 722 | 721 | 722 | $\beta_{op}$ *cis* (CH); ν (CN); ϱ ($CH_2$); ν (CS) | Amino acids [9]; fatty acid chains [3]; RSSR-, RSOSR-, $RSO_2SR$-containing molecules adsorbed on SeNP |
| 700 | 700 | 701 | 701 | 699 | 700 | ω (CO); δ (=CH); ν (CS) | Alkyl halides, carboxylic acids, and amines [2-4] |
| | | | 670 | 671 | 673 | δ (OH); δ (CCC); δ (COO) | Amino acid residues [9]; $RSO_2^-$-containing molecules adsorbed on SeNP |
| 631(s) | 630(s) | 629(s) | | | | ν (CS); δ (OH); δ (S)OH | RSH-containing molecules; RSH-, $RS^-$-, $RSO_3^-$-, RSSR-containing molecules adsorbed on SeNP |
| | | | 534 | 532 | 533 | δ (OH); ϱ ($COO^-$); backbone vibrations | RSH-, $RSO_2H$-, RSSR-containing molecules; $RSO_2H$-, $RSO_3^-$-containing molecules adsorbed on SeNP |
| 527 | 527 | 528 | 527 | 528 | 525 | δ (OH); δ ($COO^-$); backbone vibrations | Amino acid residues [9]; $RSO_3H$-, $RSO_3^-$-containing molecules; $RS^-$-, $RSO_3H$-containing molecules adsorbed on SeNP |
| 520 | 521 | 520 | 519 | 518 | 519 | $\delta_{op}$ (OH); δ ($COO^-$); ω (OH) | Amino acid residues [9]; polysaccharides [14]; $RSO_3H$-containing molecules adsorbed on SeNP |
| | 469 | 470 | 470 | 471(s) | 471(s) | δ (OH); τ ($NH_2$) | Amino acid residues [9]; $RSO_2^-$-, $RSO_3^-$-$RSO_2SR$-containing molecules; $RSO_2SR$-containing molecules adsorbed on SeNP |

| | | | | | | | |
|---|---|---|---|---|---|---|---|
| 409(s) | 408(s) | 410(s) | 412 | 415 | 411 | δ (OH); δ ($SO_3$) | Amino acid residues [9]; $RSO_2H$-containing molecules; $RSO_2H$-, $RSO_3^-$-, RSSR-containing molecules adsorbed on SeNP |

Where ν, δ, β, ϱ, τ, and ω indicate stretching, bending, deformation, rocking, twisting, and wagging, respectively; sciss, oph, as, s, and ip stand for scissoring, out of phase, asymmetric, symmetric, and in plane vibrations.

**Table S4.** ATR-FTIR absorption bands and identification of biogenic samples (Bio SeNP extract, OM, and Bio SeNP extract_w).

| **$\tilde{\nu}$ ($cm^{-1}$)** | | | **Vibrational modes** | **Identification** |
|---|---|---|---|---|
| **Bio SeNP extract** | **OM** | **Bio SeNP extract_w** | | |
| | | 3438(s) | ν (OH) | H-bonded water impurities [15] |
| | | 3387(s) | ν (OH); $\nu_{as}$ (NH) | Proteins [16] |
| | | 3349(s) | ν (OH); $\nu_s$ (NH) | Proteins [16] |
| | | 3303 | ν (OH); ν (NH) | Water; proteins (Amide A) [10] |
| 3289 | 3285 | | ν (NH) | Proteins (Amide A) [1] |
| 3200(s) | | 3220(s) | ν (CH); ν (NH) | Free amino acid residues [17] |
| | | 3145(s) | ν (CH); $\nu_{as}$ ($NH^{3+}$) | Proteins or amino acid residues [11]; $RS^-$-containing molecules adsorbed on SeNP |
| | | 3092 | $\nu_s$ ($NH^{3+}$); ν (CH) | Microbial proteins (Amide A) [10]; $S^-$ and $SO_2^-$ adsorbed on SeNP |
| 3070 | 3073 | 3065 | $\nu_s$ ($NH^{3+}$) | Proteins (Amide B) [2] |
| | | 3030 | ν (CH); $\nu_s$ ($NH^{3+}$) | Amino acid residues [9]; RSH- and $RSO_2^-$-containing molecules adsorbed on SeNP |
| 2950 | 2960 | 2956 | $\nu_{as}$ ($CH_3$) | Fatty acids [1-3] |
| 2923 | 2923 | 2925 | $\nu_{as}$ ($CH_2$) | Fatty acids [1-3] |
| 2877 | 2873 | 2872 | $\nu_s$ (CH) | Amino acids in fatty acids [3] |
| 2857 | 2851 | 2856 | $\nu_s$ ($CH_2$) | Fatty acids [1-3] |
| | | 2476 | ν (-$NH^+$) | Free amino acids [17] |
| | | 2410 | ν (-$NH^+$) | Free amino acids [17] |
| 1740 | 1739 | 1737 | ν (CO) | Ester moieties of lipids and polysaccharides [1-3] |
| 1649 | 1651 | 1652 | ν (CO); ν *cis* (HC=CH) | α-helix proteins [1-3] |

| | | | | |
|---|---|---|---|---|
| 1634(s) | 1629(s) | 1634(s) | $\nu_{as}$ ($COO^-$); β ($NH_2$); δ (NH) | β-sheet proteins [1,10] |
| 1538 | 1538 | 1548(s) | δ (NH); ν (CN) | α-helix proteins (Amide II) [1-3] |
| 1517(s) | 1511(s) | | $\delta_s$ ($NH_3^+$) | Proteins [2]; RSH-containing molecules |
| 1458 | 1457 | | $\beta_{scissoring}$ ($CH_2$); $\varrho_{as(oph)}$ (CH); $\delta_{as}$ ($CH_3$); δ (OH); ν CC(O) | Lipids, proteins and polyesters ([1,3] |
| | | 1440 | $\delta_{sciss}$ ($CH_2$); δ (OH); ν CC(O); $\nu_s$ ($COO^-$) | Polysaccharides [4]; aliphatic groups of proteins [5]; $RSO_2H$-containing molecules adsorbed on SeNP |
| 1385 | 1384 | 1385(s) | δ (CH); δ (COH); β ($CH_3$); ν CC(O); $\nu_s$ ($COO^-$); δ ($NH_2$); ν (CN) | Aldehydes; carboxylic acids; peptides [5]; aromatic amines [6] |
| | | 1329 | δ (CH); ϱ ($CH_2$); δ (OH); β C(OH) | Polysaccahrides [3-4]; RSH-containing molecules; $RS^-$-containing molecules adsorbed on SeNP |
| 1303 | 1301 | 1304 | ω ($CH_2$); ν (CN); δ ($NH_2$); δ (OH); δ (CH); $\varrho_{as(oph)}$ (CH); ν (COC); ν (CCO) | Ester moieties [1]; proteins (Amide III) [1-3]; RSH-, $RSO_2^-$-, $RSO_3H$-, RSSR-, RSOSR-containing molecules; RSH-and $RSO_2H$-, $RSO_3H$-, $RSO_3^-$-containing molecules adsorbed on SeNP; |
| 1256 | 1253(s) | 1246 | ν (CN); δ ($NH_2$) | Proteins (Amide III) [1-3] |
| 1229 | 1231 | | ν CO(H); δ (COH); ν (CO); $\nu_{as}$ ($PO_2^-$); ν (CN) | Nucleic acids [15]; amino acids [12] |
| 1173(s) | 1169 | | ν (CN); ϱ (NH); $\varrho_{as(oph)}$ (CH); ν (CO); δ (CH); δ (S)OH; ν (CC); δ (OH) | Triglyceride ester linkage; $\beta_{(1,3)}$ glycosidic bonds [7]; amino acids [9]; $RS^-$-, RSSR-containing molecules; RSH-containing molecules adsorbed on SeNP |
| 1151(s) | 1149 | 1158(s) | $\nu_{as}$ (COC); δ ($CH_2$); δ (CH); δ ($NH_2$); δ (OH); δ (S)OH; ν (CO) | Nucleic acids; $\alpha_{(1,4)}$ glycosidic bonds [7]; polysaccharide ring [8]; amino acids [9]; RSH-containing molecules |
| 1098 | 1100 | | $\nu_{as}$ (COC); ν (CC); ν (CO); δ (COH); ν $P(OH)_2$ | $\beta_{(1,4)}$ glycosidic bonds [7]; amino acids [9]; polysaccharides [4,8] |
| | | 1070 | ν (CO); ν (CC); ν (COH); δ (COC); | Polysaccharides, proteins, and polyesters [10]; amino acid residues [11]; $RSO_2^-$-containing |

| | | | | |
|---|---|---|---|---|
| | | | ϱ ($NH_3^+$); ν (SO) | molecules adsorbed on SeNP |
| 1057 | 1059 | 1052(s) | ν (CO); ϱ (CO); $\nu_s$ ($PO_2^-$); ν (CC); ν (C)OH; ν (COH); δ (COC) | Nucleic acids, phospholipids, and polysaccharides [1-4] |
| 981 | 980 | | δ ($NH_2$); δ (HNCC); ν (CO); $\nu_s$ ($PO_3^{2-}$); ν (CO); ν (CC) | Amino acids [9,12]; $\beta_{(1,3)}$ glycosidic bonds [7]; nucleic acids [10]; $RS^-$-containing molecules adsorbed on SeNP |
| | | 969(s) | δ ($NH_2$); δ (HNCC) | Amino acid residues [9]; RSH-containing molecules adsorbed on SeNP |
| 932 | 926 | 931 | δ (=CH); τ ($CH_2$); $\nu_s$ ($PO_4^{3-}$); δ (SH); ν (SO); δ ($NH_2$); δ ($CH_2$) | Alkyl halides, carboxylic acids; amines; $\alpha_{(1,3)}$ glycosidic bonds [7]; amino acid residues [9]; nucleic acids [4]; RSH-, RSSR-, $RSO_2SR$-containing molecules; RSSR-, RSOSR-, $RSO_2SR$-containing molecules adsorbed on SeNP |
| 899 | 894 | 897 | δ ($NH_2$); ν (COC); ν (CC); ν (CN); δ ($CH_2$) | $\beta_{(1,4)}$ glycosidic bonds [7]; amino acid residues [9]; $RSO_3H$-, $RSO_3^-$-containing molecules; $RS^-$-, $RSO_3^-$-, RSSR-, RSOSR-containing molecules adsorbed on SeNP |
| | | 857(s) | δ ($NH_2$); ν (CC); ν (CN) | Amino acid residues [9]; $RSO_2H$-containing molecules adsorbed on SeNP |
| 824(s) | | | δ ($NH_2$); ω ($COO^-$); δ ($CH_2$); δ (OH) | RSH-, $RSO_3H$-, $RSO_3^-$-, RSSR-, $RSO_2SR$-containing molecules; RSH-, $RSO_2H$-, $RSO_2^-$-, $RSO_3H$-, $RSO_3^-$-, RSOSR-, $RSO_2SR$-containing molecules adsorbed on SeNP |
| 806 | 811 | | ν SO(H); δ (OH) $\delta_{op}$ (HOCC); ν (PO) | Amino acids [9]; nucleic acids [13]; RSH-, $RS^-$-, RSSR-containing molecules; $RS^-$-, RSOSR-containing molecules adsorbed on SeNP |
| | | 790 | ν SO(H); ϱ ($CH_2$) | RSH-containing molecules; $RSO_2H$-containing molecules adsorbed on SeNP |
| 769 | | 768 | ν C(COOH); δ ($NH_2$); ϱ ($CH_2$); δ (HNC); δ (CCH); δ (OH) | Amino acids [9]; RSH-, RSSR-, RSOSR-, $RSO_2SR$-containing molecules; $RSO_3^-$-containing molecules adsorbed on SeNP |
| 740 | 738 | | δ ($NH_2$)C(COOH); | Amino acids [9]; RSH-, $RS^-$-, $RSO_2H$-, $RSO_2^-$-, |

| | | | | |
|---|---|---|---|---|
| | | | ϱ ($CH_2$); δ (OH) | $RSO_2SR$-containing molecules adsorbed on SeNP |
| 714(s) | 717 | 718(s) | $\beta_{op}$ *cis* (CH); ν (CN); ϱ ($CH_2$); ν (CS) | Amino acids [9]; fatty acid chains [3]; RSSR-, RSOSR-, $RSO_2SR$-containing molecules adsorbed on SeNP |
| 700 | 700 | 697 | ω (CO); δ (=CH); ν (CS) | Alkyl halides, carboxylic acids and amines [1-4] |
| | | 672 | δ (OH); δ (CCC); δ (COO) | Amino acid residues [9]; $RSO_2^-$-containing molecules adsorbed on SeNP |
| 659 | 660 | 652(s) | δ (OH); $\delta_{op}$ (HOCC) | Amino acid residues [9]; $RSO_2H$-, RSOSR-containing molecules adsorbed on SeNP |
| | | 641 | δ (OH); $\delta_{op}$ (HOCC) | Amino acid residues [9]; $RS^-$-, $RSO_2^-$-, and $RSO_3H$-containing molecules adsorbed on SeNP |
| 625 | | 626 | ν (CS); δ (OH); δ (S)OH | RSH-containing molecules; RSH-, $RS^-$-, $RSO_3^-$-, RSSR-containing molecules adsorbed on SeNP |
| 604 | | 604 | δ (OH); $\delta_{op}$ ($NH_2$) | Amino acid residues [9]; $RSO_2^-$-containing molecules |
| | | 595 | δ (OH) | $RSO_2^-$- and $RSO_3H$-containing molecules adsorbed on SeNP |
| | | 588 | δ ($NH_2$); δ ($COO^-$) | Amino acid residues [9]; $RSO_3^-$- containing molecules adsorbed on SeNP |
| | | 577 | δ ($COO^-$); $\delta_{op}$ (OH) | Amino acid residues [9]; $RSO_2^-$- containing molecules adsorbed on SeNP |
| 566 | | | δ (OH); δ ($NH_2$); δ (OCOH); backbone vibrations | Amino acid residues [9]; RSOSR-containing molecules; $RS^-$-, $RSO_2^-$-, $RSO_2SR$-containing molecules adsorbed on SeNP |
| | | 555 | δ (OH); δ ($NH_2$); δ (OCOH) | Amino acid residues [9]; RSH-containing molecules adsorbed on SeNP |
| | 534 | 536 | δ (OH); ϱ ($COO^-$); backbone vibrations | RSH-, $RSO_2H$-, RSSR-containing molecules; $RSO_2H$-, $RSO_3^-$-containing molecules adsorbed on SeNP |
| 524 | | 524(s) | δ (OH); δ ($COO^-$); backbone vibrations | Amino acid residues [9]; $RSO_3H$-, $RSO_3^-$-containing molecules; $RS^-$-, $RSO_3H$-containing molecules adsorbed on SeNP |
| | | 514 | $\delta_{op}$ (OH); ω (OH); δ ($COO^-$) | Amino acid residues [9]; $RSO_3H$-containing |

| | | | | |
|---|---|---|---|---|
| | | | | molecules adsorbed on SeNP |
| | | 500(s) | δ ($SO_2$) | $RSO_3^-$-containing molecules adsorbed on SeNP |
| | | 473 | δ ($SO_2$); δ (OH) | $RSO_2H$- and $RSO_3H$-containing molecules adsorbed on SeNP |
| 464 | 464 | 464 | δ (OH); τ ($NH_2$) | Amino acid residues [9]; $RSO_2^-$-, $RSO_3^-$-$RSO_2SR$-containing molecules; $RSO_2SR$-containing molecules adsorbed on SeNP |
| | | 456 | δ (CNN) | RSH-containing molecules |
| | | 435(s) | δ SO(OH) | $RSO_3H$-containing molecules adsorbed on SeNP |
| 408 | | 409 | δ (OH); δ ($SO_3$) | Amino acid residues [9]; $RSO_2H$-containing molecules; $RSO_2H$-, $RSO_3^-$-, RSSR-containing molecules adsorbed on SeNP |
| | | 400(s) | δ ($SO_2$) | $RSO_2H$- containing molecules adsorbed on SeNP |
| | | 386 | | |
| 375 | | 369 | δ (NCC); δ (CCCO); δ (SH); δ (CCN); τ ($NH_3$); δ ($NH_2$); backbone vibrations | Amino acid residues [9]; RSH-, and $RS^-$-, RSSR-containing molecules; $RSO_2^-$-containing molecules adsorbed on SeNP |
| | | 358 | δ (NCC); δ (CCCO); δ (SH) | Amino acid residues [9]; $RSO_2^-$-containing molecules adsorbed on SeNP |
| | | 347 | δ ($NH_2$) | $RSO_2^-$-containing molecules adsorbed on SeNP |
| 337 | | 337 | δ ($NH_2$); δ (OH); $\delta_{ip}$ (CCC) | Amino acid residues [9]; RSOSR-, $RSO_2SR$-containing molecules; RSH-, RSSR-, RSOSR-, $RSO_2SR$-containing molecules adsorbed on SeNP |
| | | 325 | δ ($NH_2$); τ ($COO^-$); backbone vibrations | Amino acid residues [9]; $RSO_3H$-, RSOSR-, $RSO_2SR$-containing molecules; RSH-, $RSO_2^-$-, RSSR-, $RSO_2SR$-containing molecules adsorbed on SeNP |
| 316 | | 316 | δ ($NH_2$); τ ($COO^-$); β (CN); δ (SH) | Amino acid residues [9]; $RSO_3H$-, RSOSR-, $RSO_2SR$-containing molecules; RSH-, $RSO_2^-$-, RSSR-, $RSO_2SR$-containing molecules adsorbed |

| | | | |
|---|---|---|---|
| | | | on SeNP |
| | 305 | δ (SH); τ ($COO^-$) | Amino acid residues [9]; RSH-containing molecules adsorbed on SeNP |
| 294 | 290 | ν (S)OH; δ ($NH_2$); δ (CCC); backbone vibrations | Amino acid residues [9]; RSH-, $RSO_2^-$-, $RSO_3H$-, $RSO_3^-$-, RSOSR-, $RSO_2SR$-containing molecules; $RSO_2^-$- and $RSO_2SR$-containing molecules adsorbed on SeNP |
| | 280 | δ ($NH_2$); τ ($NH_2$) | Amino acid residues [9]; $RS^-$-containing molecules adsorbed on SeNP |
| | 272 | $δ_{ip}$ (CCN); δ (SH); backbone vibrations; δ ($NH_2$) | RSH-, $RS^-$-, $RSO_3^-$-, RSSR-, RSOSR-, $RSO_2SR$-containing molecules; RSH-, $RSO_2^-$-, RSSR-, RSOSR-, $RSO_2SR$-containing molecules adsorbed on SeNP |
| 265 | 266 | τ ($NH_2$); $Se_8$ vibration; backbone vibrations | RSH-, $RS^-$-, $RSO_2H$-, $RSO_2^-$-, $RSO_3H$-, $RSO_3^-$-, RSSR-, RSOSR-, $RSO_2SR$-containing molecules adsorbed on SeNP |
| | 260 | $Se_8$ vibration | RSH-, $RSO_2H$-, $RSO_2^-$-, $RSO_3H$-, $RSO_3^-$-, RSSR-, RSOSR-, $RSO_2SR$-containing molecules |
| 249 | 249 | $Se_8$ vibration | $RS^-$-, $RSO_2^-$-, $RSO_3H$-, $RSO_3^-$-, RSOSR-, $RSO_2SR$-containing molecules adsorbed on SeNP |

Where ν, δ, β, ϱ, τ, and ω indicate stretching, bending, deformation, rocking, twisting, and wagging, respectively; sciss, oph, as, s, and ip stand for scissoring, out of phase, asymmetric, symmetric, and in plane vibrations.

nanomaterials

MDPI

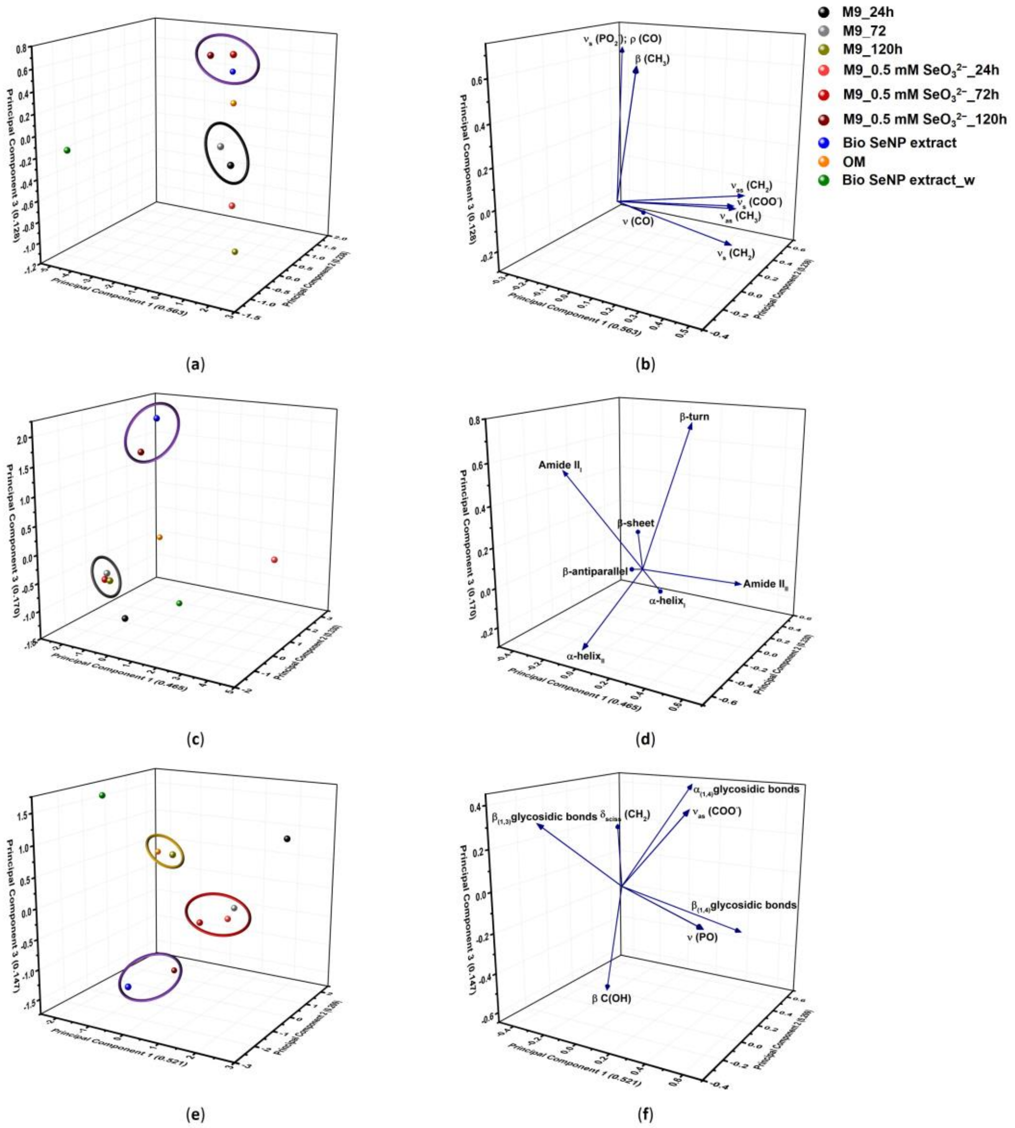


**Figure S3.** Representation of (**a, c,** and **e**) score and (**b, d,** and **f**) loading plots obtained by performing PCA on IR contributions referring to (**a-b**) lipids, (**c-d**) proteins, and (**e-f**) polysaccharides of *Micrococcus* sp. cells incubated in the presence/absence of $SeO_3^{2-}$ and the derived Bio SeNP extracts, alongside OM.

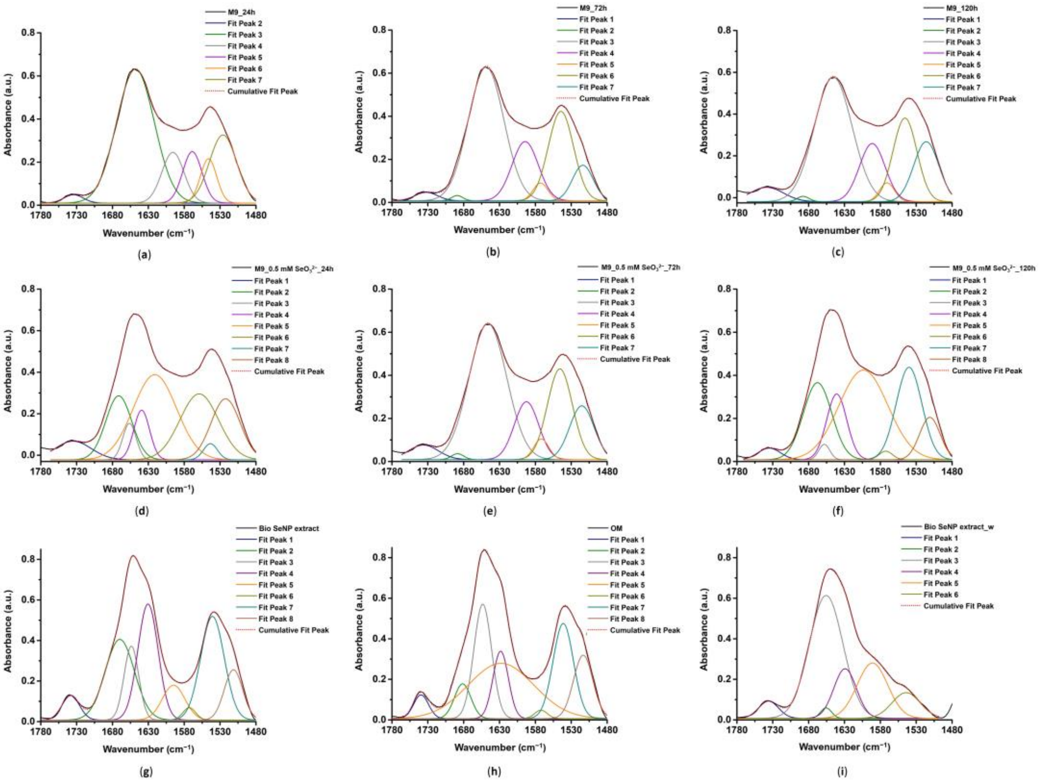


**Figure S4.** ATR-FTIR spectral deconvolution performed on the 1780-1480 cm$^{-1}$ region for *Micrococcus* sp. cells incubated for (**a, d**) 24h, (**b, e**) 72h, and (**c, f**) 120h with either (**a-c**) glucose or (**d-f**) glucose and $SeO_3^{2-}$, (**g**) Bio SeNP extract, (**h**) OM, and (**i**) Bio SeNP extract_w.

**Table S5.** Deconvolution of ATR-FTIR spectra of *Micrococcus* sp. unchallenged cells in the 1780-1480 cm$^{-1}$ region.

| M9_24h | w | A | M9_72h | w | A | M9-120h | w | A | Vibrational mode |
|---|---|---|---|---|---|---|---|---|---|
| 1734 | 26.74 | 1.293 | 1732 | 31.42 | 1.554 | 1738 | 46.56 | 4.008 | ν (CO) |
| | | | 1670 | 15.72 | 0.484 | 1675 | 16.73 | 0.536 | δ (NH); ν (CO) |
| 1648 | 52.25 | 40.86 | 1649 | 49.49 | 38.51 | 1645 | 52.54 | 39.27 | ν (CO) |
| 1596 | 30.34 | 9.116 | 1594 | 36.71 | 12.65 | 1591 | 35.72 | 12.46 | $\delta_{as}$ ($NH_3^+$) |
| 1569 | 26.88 | 8.031 | 1572 | 20.61 | 2.137 | 1570 | 20.84 | 2.352 | $\nu_{as}$ ($COO^-$) |
| 1546 | 22.75 | 5.852 | 1544 | 35.25 | 18.34 | 1545 | 33.39 | 16.74 | $\delta_s$ (NH); ν (CN) |
| 1526 | 37.85 | 14.98 | | | | | | | |
| | | | 1514 | 28.94 | 5.983 | 1516 | 35.08 | 12.63 | $\delta_s$ ($NH_3^+$) |

Where ν, δ, as, and s indicates stretching, bending, asymmetric, and symmetric vibrations, respectively.

**Table S6.** Deconvolution of ATR-FTIR spectra of *Micrococcus* sp. cells incubated with $SeO_3^{2-}$ in the 1780-1480 cm$^{-1}$ region.

| M9_0.5 mM $SeO_3^{2-}$_24h | w | A | M9_0.5 mM $SeO_3^{2-}$_72h | w | A | M9_0.5 mM $SeO_3^{2-}$_120h | w | A | Vibrational mode |
|---|---|---|---|---|---|---|---|---|---|
| 1734 | 50.54 | 5.681 | 1736 | 41.63 | 3.533 | 1735 | 31.25 | 2.116 | ν (CO) |
| | | | 1689 | 16.91 | 0.581 | | | | |
| 1671 | 36.16 | 13.96 | | | | 1668 | 39.56 | 17.73 | δ (NH); ν (CO) |

| | | | | | | | | | |
|---|---|---|---|---|---|---|---|---|---|
| 1656 | 18.63 | 4.094 | | | | 1658 | 15.35 | 1.373 | ν (CO) |
| | | | 1646 | 52.45 | 41.53 | | | | ν (CO) |
| 1639 | 22.09 | 6.615 | | | | 1640 | 28.11 | 10.74 | ν (CO); δ (NH) |
| 1621 | 60.29 | 30.97 | | | | | | | ν (CO); δ (NH) |
| | | | 1592 | 33.90 | 11.40 | 1603 | 68.29 | 35.61 | $\delta_{as}$ ($NH_3^+$) |
| | | | 1572 | 21.00 | 2.524 | 1572 | 18.70 | 0.933 | $\nu_{as}$ ($COO^-$) |
| 1559 | 53.90 | 21.44 | | | | | | | $\delta_s$ (NH); ν (CN) |
| 1543 | 19.64 | 1.915 | 1546 | 34.88 | 18.39 | 1540 | 35.92 | 19.32 | $\delta_s$ (NH); ν (CN) |
| 1522 | 40.69 | 14.98 | | | | | | | $\delta_s$ (NH); ν (CN) |
| | | | 1515 | 34.07 | 10.659 | 1511 | 28.69 | 7.073 | $\delta_s$ ($NH_3^+$); $\delta_s$ (NH); ν (CN) |

Where ν, δ, as, and s indicates stretching, bending, asymmetric, and symmetric vibrations, respectively.

**Table S7.** Deconvolution of ATR-FTIR spectra of biogenic SeNP extracts and OM in the 1780-1480 $cm^{-1}$ region.

| Bio SeNP extract | w | A | OM | w | A | Bio SeNP extract_w | w | A | Vibrational mode |
|---|---|---|---|---|---|---|---|---|---|
| 1738 | 24.22 | 3.675 | 1739 | 20.72 | 3.033 | 1735 | 27.61 | 2.950 | ν (CO) |
| 1670 | 40.74 | 20.29 | 1681 | 22.95 | 4.924 | | | | δ (NH); ν (CO) |
| 1654 | 20.36 | 9.292 | 1653 | 24.74 | 17.49 | 1655 | 15.22 | 0.985 | ν (CO) |
| | | | | | | 1650 | 47.01 | 35.70 | ν (CO) |
| 1630 | 28.19 | 20.21 | 1628 | 21.40 | 8.90 | 1629 | 31.53 | 9.670 | ν (CO); δ (NH) |
| | | | 1625 | 91.94 | 31.37 | | | | ν (CO); δ (NH) |
| 1595 | 31.53 | 6.782 | | | | 1590 | 42.53 | 14.54 | $\delta_{as}$ ($NH_3^+$) |
| 1573 | 17.79 | 1.354 | 1572 | 17.60 | 0.909 | | | | $\nu_{as}$ ($COO^-$) |
| 1540 | 32.89 | 21.06 | 1541 | 28.49 | 16.75 | 1544 | 39.31 | 6.246 | $\delta_s$ (NH); ν (CN) |
| 1511 | 25.76 | 8.000 | 1513 | 27.07 | 10.61 | | | | $\delta_s$ ($NH_3^+$) |

Where ν, δ, as, and s indicates stretching, bending, asymmetric, and symmetric vibrations, respectively.

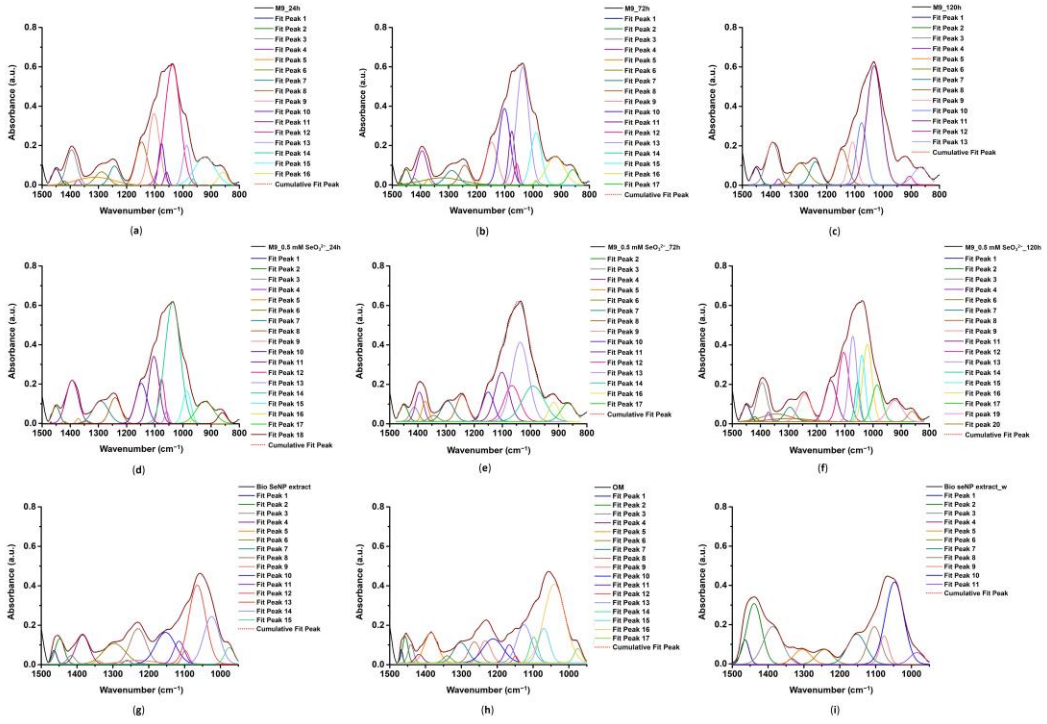

**Figure S5.** ATR-FTIR spectral deconvolution performed on the 1500-800 $cm^{-1}$ region for *Micrococcus* sp. cells incubated for (**a, d**) 24h, (**b, e**) 72h, and (**c, f**) 120h with either (**a-c**) glucose or (**d-f**) glucose and $SeO_3^{2-}$, (**g**) Bio SeNP extract, (**h**) OM, and (**i**) Bio SeNP extract_w.

**Table S8.** Deconvolution of ATR-FTIR spectra of *Micrococcus* sp. unchallenged cells in the 1500-800 cm$^{-1}$ region.

| M9_24h | w | A | M9_72h | w | A | M9_120h | w | A | Vibrational mode |
|---|---|---|---|---|---|---|---|---|---|
| | | | 1468 | 5.852 | 0.073 | | | | $\delta$ ($CH_2$); $\delta$ ($CH_3$) |
| 1450 | 28.48 | 2.977 | 1450 | 24.19 | 2.377 | 1451 | 28.14 | 3.032 | $\delta_{sciss}$ ($CH_2$); $\delta$ (OH); $\nu$ CC(O); $\nu_s$ ($COO^-$) |
| 1420 | 16.65 | 0.455 | 1421 | 22.14 | 0.927 | 1420 | 10.16 | 0.067 | $\nu_s$ ($COO^-$) |
| 1395 | 37.14 | 8.421 | 1394 | 35.58 | 7.734 | 1392 | 45.15 | 12.13 | $\delta$ (CH); $\delta$ (OH); $\delta$ (COH); $\beta$ ($CH_3$); $\nu$ (CN) |
| 1371 | 15.11 | 0.558 | 1371 | 14.90 | 0.549 | 1371 | 14.62 | 0.539 | $\beta$ ($CH_3$) |
| | | | | | | 1340 | 18.93 | 0.337 | $\beta$ C(OH); $\delta$ (OH); $\delta$ (CH) |
| 1312 | 130.1 | 6.811 | 1322 | 1318.8 | 6.297 | | | | |
| 1292 | 46.68 | 4.016 | 1288 | 47.22 | 4.319 | 1294 | 56.32 | 7.672 | $\omega$ ($CH_2$); $\varrho_{as(oph)}$ (CH); $\nu$ (COC); $\nu$ (CCO); $\delta$ (OH) |
| 1242 | 36.05 | 4.437 | 1242 | 36.21 | 4.536 | 1240 | 38.43 | 5.642 | $\beta$ (NH); $\nu_{as}$ ($PO_2^-$) |
| 1146 | 41.14 | 11.37 | 1147 | 40.83 | 11.11 | 1146 | 40.53 | 9.179 | $\nu_{as}$ (COC); $\delta$ ($CH_2$); $\delta$ (CH); $\delta$ ($NH_2$) |
| 1102 | 38.27 | 17.52 | 1101 | 39.47 | 19.21 | 1108 | 29.06 | 7.899 | $\nu_{as}$ (COC); $\nu$ (CC); $\nu$ (CO); $\delta$ (COH); $\nu$ $P(OH)_2$ |
| 1076 | 20.93 | 5.60 | 1075 | 21.75 | 7.435 | 1076 | 34.98 | 13.76 | $\nu$ (CO); $\nu$ (CC); $\nu$ (COH); $\delta$ (COC); $\varrho$ ($NH_3^+$) |
| 1058 | 15.53 | 1.319 | 1058 | 17.02 | 2.304 | | | | $\nu$ (CC); $\nu$ (CO); $\delta$ (COH); $\nu_s$ ($PO_2^-$) |
| 1037 | 53.58 | 41.26 | 1037 | 43.41 | 32.61 | 1032 | 52.90 | 39.65 | $\nu$ (PO); $\nu$ (SH); $\nu$ (SO) |
| 987 | 27.15 | 6.935 | 989 | 10.20 | 0.283 | | | | $\delta$ ($NH_2$); $\delta$ (HNCC); $\nu$ (CO); $\nu_s$ ($PO_2^-$); $\nu$ (CO); $\nu$ (CC) |
| | | | 985 | 37.81 | 12.57 | | | | $\delta$ ($NH_2$); $\delta$ (HNCC); $\nu$ (CO); $\nu_s$ ($PO_2^-$); $\nu$ (CO); $\nu$ (CC) |
| 964 | 15.47 | 0.610 | | | | | | | $\delta$ ($NH_2$); $\delta$ (HNCC) |
| 922 | 71.42 | 12.96 | 920 | 62.20 | 11.31 | | | | $\delta$ (=CH); $\tau$ ($CH_2$); $\nu_s$ ($PO_4^{3-}$); $\delta$ ($NH_2$); $\delta$ ($CH_2$) |
| | | | | | | 906 | 24.13 | 1.3 | |
| 858 | 33.04 | 2.797 | 859 | 35.32 | 3.427 | 867 | 54.04 | 5.896 | $\delta$ ($NH_2$); $\nu$ (CC); $\nu$ (CN) |

Where $\nu$, $\delta$, $\beta$, $\varrho$, and $\omega$ indicate stretching, bending, deformation, rocking, and wagging, respectively; sciss, oph, as, s, and ip stand for scissoring, out of phase, asymmetric, symmetric, and in plane vibrations.

**Table S9.** Deconvolution of ATR-FTIR spectra of *Micrococcus* sp. cells incubated with $SeO_3^{2-}$ in the 1500-800 $cm^{-1}$ region.

| M9_0.5 mM $SeO_3^{2-}$_24h | w | A | M9_0.5 mM $SeO_3^{2-}$_72h | w | A | M9_0.5 mM $SeO_3^{2-}$_120h | w | A | Vibrational mode |
|---|---|---|---|---|---|---|---|---|---|
| 1469 | 4.836 | 0.053 | | | | | | | δ ($CH_2$); δ ($CH_3$) |
| 1451 | 26.25 | 2.894 | 1450 | 28.95 | 3.056 | 1450 | 26.70 | 2.896 | $\delta_{sciss}$ ($CH_2$); δ (OH); ν CC(O); $\nu_s$ ($COO^-$) |
| 1421 | 13.07 | 0.170 | | | | | | | $\nu_s$ ($COO^-$) |
| | | | 1413 | 29.19 | 2.643 | 1415 | 17.65 | 0.570 | $\nu_s$ ($COO^-$) |
| 1393 | 43.29 | 11.93 | 1394 | 28.92 | 11.368 | 1390 | 35.80 | 8.879 | δ (CH); δ (OH); δ (COH); β ($CH_3$); ν (CN) |
| 1371 | 14.04 | 0.461 | 1373 | 22.64 | 2.917 | 1372 | 15.90 | 0.952 | β ($CH_3$); δ (CH); δ (OH) |
| 1339 | 18.35 | 0.308 | 1344 | 33.63 | 1.301 | 1342 | 103.3 | 5.042 | β C(OH); δ (OH); δ (CH) |
| 1292 | 55.53 | 7.941 | 1294 | 44.83 | 5.786 | 1303 | 39.41 | 4.330 | ω ($CH_2$); $\varrho_{as(oph)}$ (CH); ν (COC); ν (CCO); δ (OH) |
| | | | | | | 1276 | 136.2 | 2.765 | ν (CO); δ (S)OH; δ ($NH_2$); δ ($CH_2$); δ (OH); $\nu_{as}$ ($PO_2^-$) |
| 1241 | 38.92 | 6.445 | 1242 | 39.86 | 6.772 | 1243 | 43.60 | 7.644 | β (NH); $\nu_{as}$ ($PO_2^-$) |
| | | | 1220 | 0.023 | 232.2 | 1223 | 0.202 | 3.300 | ν CO(H) δ (COH); ν (CO); $\nu_{as}$ ($PO_2^-$); ν (CN); δ (S)OH; δ ($CH_2$) |
| 1206 | 11.12 | 0.078 | | | | | | | ν (CO); δ ($CH_2$); δ (CH); δ (OH) |
| 1148 | 40.86 | 10.46 | 1150 | 40.06 | 7.479 | 1150 | 41.20 | 10.74 | $\nu_{as}$ (COC); δ ($CH_2$); δ (CH); δ ($NH_2$) |
| 1102 | 36.70 | 15.72 | 1101 | 50.11 | 15.68 | 1104 | 37.30 | 16.43 | $\nu_{as}$ (COC); ν (CC); ν (CO); δ (COH); ν $P(OH)_2$ |
| 1075 | 21.97 | 6.195 | 1066 | 55.63 | 12.69 | 1072 | 27.65 | 14.99 | ν (CO); ν (CC); ν (COH); δ (COC); ϱ ($NH_3^+$); ν (SO) |
| 1058 | 15.22 | 1.191 | | | | 1055 | 18.59 | 4.663 | ν (CC); ν (CO); δ (COH); ν (SO); $\nu_s$ ($PO_2^-$) |
| 1037 | 53.65 | 41.51 | 1036 | 59.66 | 29.99 | 1040 | 23.48 | 9.951 | ν (PO); ν (SH); ν (SO) |
| | | | | | | 1020 | 32.36 | 15.89 | ν (POC); δ ($NH_2$) |
| 986 | 28.81 | 5.980 | 990 | 77.64 | 17.44 | 985 | 35.91 | 8.389 | δ ($NH_2$); δ (HNCC); ν (CO); $\nu_s$ ($PO_2^-$); ν (CO); ν (CC) |
| 963 | 13.96 | 0.381 | | | | | | | δ ($NH_2$); δ (HNCC) |
| | | | | | | 940 | 240.6 | 3.169 | |
| 922 | 62.24 | 8.829 | 917 | 34.48 | 4.227 | 921 | 51.53 | 6.940 | δ (=CH); τ ($CH_2$); $\nu_s$ ($PO_4^{3-}$); δ (SH); ν (SO); δ ($NH_2$); δ ($CH_2$) |
| 860 | 29.97 | 2.098 | 866 | 50.34 | 5.842 | 861 | 31.44 | 2.060 | δ ($NH_2$); ν (CC); ν (CN) |

Where ν, δ, β, ϱ, and ω indicate stretching, bending, deformation, rocking, and wagging, respectively; sciss, oph, as, s, and ip stand for scissoring, out of phase, asymmetric, symmetric, and in plane vibrations.

**Table S10.** Deconvolution of ATR-FTIR spectra of biogenic SeNP extracts and OM in the 1500-950 cm$^{-1}$ region.

| Bio SeNP extract | w | A | OM | w | A | Bio SeNP extract_w | w | A | Vibrational mode |
|---|---|---|---|---|---|---|---|---|---|
| 1464 | 14.59 | 1.263 | 1468 | 7.498 | 0.624 | 1464 | 21.87 | 3.420 | δ (CH$_2$); δ (CH$_3$) |
| | | | 1457 | 14.99 | 2.491 | | | | β$_{sciss}$ (CH$_2$); δ$_{as}$ (CH$_3$); ϱ$_{as(oph)}$ (CH); δ (OH); ν CC(O) |
| 1448 | 24.84 | 4.015 | 1441 | 15.65 | 1.778 | 1438 | 41.79 | 16.19 | δ (OH); ν CC(O) |
| 1416 | 22.36 | 1.205 | 1418 | 21.19 | 1.140 | | | | ν$_s$ (COO$^-$) |
| 1385 | 36.38 | 6.738 | 1385 | 36.40 | 6.969 | 1386 | 52.58 | 12.55 | δ (CH); δ (OH); δ (COH); β (CH$_3$); ν (CN) |
| 1342 | 18.11 | 0.402 | 1339 | 23.07 | 1.023 | 1333 | 21.58 | 0.777 | β C(OH); δ (OH); δ (CH) |
| 1296 | 61.55 | 8.045 | 1302 | 38.94 | 5.022 | 1304 | 46.08 | 4.393 | ω (CH$_2$); ϱ$_{as(oph)}$ (CH); ν (COC); ν (CCO); δ (OH) |
| 1260 | 16.38 | 0.395 | 1264 | 30.58 | 4.070 | | | | ν (CO); δ (S)OH |
| | | | | | | 1242 | 44.49 | 4.208 | β (NH); ν$_{as}$ (PO$_2^-$) |
| 1229 | 44.80 | 10.08 | 1233 | 30.75 | 4.293 | | | | ν CO(H) δ (COH); ν (CO); ν$_{as}$ (PO$_2^-$); ν (CN); δ (S)OH; δ (CH$_2$) |
| 1226 | 60.58 | 2.126 | | | | | | | ν CO(H) δ (COH); ν (CO); ν$_{as}$ (PO$_2^-$); ν (CN); δ (S)OH; δ (CH$_2$) |
| | | | 1211 | 55.61 | 8.460 | | | | ν (CO); δ (CH$_2$); δ (CH); δ (OH) |
| | | | 1166 | 24.27 | 2.758 | | | | ν (CN); ϱ$_{as(oph)}$ (CH); ϱ (NH); ν (CO); δ (S)OH; δ (CH); ν (CC) |
| 1153 | 55.17 | 11.09 | 1150 | 12.71 | 0.564 | 1152 | 51.15 | 9.825 | ν$_{as}$ (COC); δ (CH$_2$); δ (CH); δ (NH$_2$) |
| | | | 1125 | 40.63 | 9.824 | | | | δ$_{ip}$ (OH) |
| 1115 | 30.06 | 4.358 | | | | | | | ϱ (CNH$_3$); ω (CH$_2$); ν$_{as}$ (PO$_2^-$) |
| 1098 | 18.85 | 1.602 | 1098 | 22.96 | 3.732 | 1103 | 37.22 | 8.934 | ν$_{as}$ (COC); ν (CC); ν (CO); δ (COH); ν P(OH)$_2$ |
| 1065 | 49.97 | 23.56 | 1069 | 31.27 | 6.810 | 1076 | 25.31 | 4.550 | ν (CO); ν (CC); ν (COH); δ (COC); ϱ (NH$_3^+$); ν (SO); ν$_s$ (PO$_2^-$) |
| | | | 1040 | 58.44 | 29.24 | 1047 | 50.59 | 26.58 | ν (PO); ν (SH); ν (SO) |
| 1024 | 44.07 | 13.31 | | | | | | | ν (POC) |
| 975 | 26.12 | 2.751 | 974 | 21.58 | 1.949 | 984 | 39.18 | 2.902 | δ (NH$_2$); δ (HNCC); ν (CO); ν$_s$ (PO$_2^-$); ν (CO); ν (CC) |

Where ν, δ, β, ϱ, and ω indicate stretching, bending, deformation, rocking, and wagging, respectively; sciss, oph, as, s, and ip stand for scissoring, out of phase, asymmetric, symmetric, and in plane vibrations.

## References


1. Tugarova, A.V.; Mamchenkova, P.V.; Dylatova, Y.A.; Kamnev, A.A. FTIR and Raman spectroscopic studies of selenium nanoparticles synthesized by the bacterium *Azospirillum thiophilum*. *Spectrochim. Acta A. Mol. Biomol. Spectrosc.* **2018**, *192*, 458–463. https://doi.org/10.1016/j.saa.2017.11.050
2. Faghihzadeh, F.; Anaya, N.M.; Schifman, L.A.; Oyanedel-Craver, V. Fourier transform infrared spectroscopy to assess molecular-level changes in microorganisms exposed to nanoparticles. *Nanotechnol. Environ. Eng.* **2016**, *1*, 1. https://doi.org/10.1007/s41204-016-0001-8
3. Lasch, P.; Naumann, D. Infrared spectroscopy in microbiology. In: *Encyclopedia of Analytical Chemistry*. Meyers, R.A., Ed.; Wiley Online Library, 2015. https://doi.org/10.1002/9780470027318.a0117.pub2
4. Jiang, W.; Saxena, A.; Song, B.; Ward, B.B.; Beveridge, T.J.; Myneni, S.C.B. Elucidation of functional groups on Gram-positive and Gram-negative bacterial surfaces using Infrared spectroscopy. *Langmuir* **2004**, *20*, 11433–11442. https://doi.org/10.1021/la049043+
5. Buszewski, B.; Dziubakiewicz, E.; Pomastowski, P.; Hrynkiewicz, K.; Ploszaj-Pyrek, J.; Talik, E.; Kramer, M.; Albert, K. Assignment of functional groups in Gram-positive bacteria. *J. Anal. Bioanal. Tech.* **2015**, *6*, 1. https://doi.org/10.4172/2155-9872.1000232
6. Otari, S.V.; Patil, R.M.; Ghosh, S.J.; Thorat, N.D.; Pawar, S.H. Intracellular synthesis of silver nanoparticle by actinobacteria and its antimicrobial activity. *Spectrochim. Acta A Mol. Biomol. Spectrosc.* **2015**, *136*, 1175-1180. http://dx.doi.org/10.1016/j.saa.2014.10.003
7. Nikonenko, N.A.; Buslov, D.K., Sushko, N.J.; Zhbankov, R.G. Investigation of stretching vibrations of glycosidic linkages in disaccharides and polysaccharides with use of IR spectra deconvolution. *Biopolymers* 2000, 57, 257–262. https://doi.org/10.1002/1097-0282(2000)57:4<257::AID-BIP7>3.0.CO;2-3
8. Nadtochenko, V.A.; Rincon, A.G.; Stanca, S.E., Kiwi, J. Dynamics of *E. coli* membrane cell peroxidation during $TiO_2$ photocatalysis studied by ATR-FTIR spectroscopy and AFM microscopy. *J. Photochem. Photobiol. A Chem.* **2005**, *169*, 131–137. https://doi.org/10.1016/j.jphotochem.2004.06.011
9. Mohamed, M.E.; Mohammed, A.M.A. Experimental and computation vibration study of amino acids. *Inter. Lett. Chem. Phys. Astr.* **2013**, *10*, 1–17. https://doi.org/10.18052/www.scipress.com/ILCPA.15.1
10. Kamnev, A.A.; Mamchenkova, P.V.; Dylatova, Y.A.; Tugarova, A.V. FTIR spectroscopic studies of selenite reduction by cells of the rhizobacterium *Azospirillum brasilense* Sp7 and the formation of selenium nanoparticles. *J. Mol. Struct.* **2017**, *1140*, 106–112. http://dx.doi.org/10.1016/j.molstruc.2016.12.003
11. Kurihara, T.; Noda, Y.; Takegoshi, K. Capping structure of ligand-cysteine on CdSe magic-sized clusters. *ACS Omega* **2019**, *4*, 3476-3483. https://doi.org/10.1021/acsomega.8b02752
12. Barth, A. The infrared absorption of amino acid side chains. *Progr. Biophys. Mol. Biol.* **2000**, *74*, 141–173. https://doi.org/10.1016/S0079-6107(00)00021-3
13. Garip, S.; Gozen, A.C.; Severcan, F. Use of Fourier transform infrared spectroscopy for rapid comparative analysis of *Bacillus* and *Micrococcus* isolates. *Food Chem.* **2009**, *113*, 1301–1307. https://doi.org/10.1016/j.foodchem.2008.08.063
14. Wiercigroch, E.; Szafraniec, E.; Czamara, K.; Pacia, M.Z.; Majzner, K.; Kochan, K.; Kaczor, A.; Baranska, M.; Malek, K. Raman and infrared spectroscopy of carbohydrates: a review. *Spectrochim. Acta A Mol. Biomol. Spectrosc.* **2017**, *185*, 317–335. http://dx.doi.org/10.1016/j.saa.2017.05.045
15. Kamnev, A.A. FTIR spectroscopic studies of bacterial cellular responses to environmental factors, plant-bacterial interactions and signalling. *Spectroscopy* **2008**, *22*, 83–85. https://doi.org/10.3233/SPE-2008-0329
16. Tanaka, I.; Tellez, C.; Oliveira, R.P.D.; Fortes, S.A.; Soto, C.A.T. Analysis of the Fourier Transform Infrared (Attenuated Total Reflection) spectra in the study of multi-sensitive bacteria of the genus *Klebsiella* sp. using a fine copper sheet as sensor. *J. Phys. Chem. Res.* **2020**, *2*, 115. https://doi.org/10.36266/JPCR/115
17. Heacock, R.A.; Marion, L. The Infrared spectra of secondary amines and their salts. *Can. J. Chem.* **1956**, 1782–1795.
